# Tracheostomy timing and outcome in critically ill patients with stroke: a meta-analysis and meta-regression

**DOI:** 10.1186/s13054-023-04417-6

**Published:** 2023-04-01

**Authors:** Lavienraj Premraj, Christopher Camarda, Nicole White, Daniel Agustin Godoy, Brian H. Cuthbertson, Patricia R. M. Rocco, Paolo Pelosi, Chiara Robba, Jose I. Suarez, Sung-Min Cho, Denise Battaglini

**Affiliations:** 1 Griffith University School of Medicine, Gold Coast, Queensland, Australia; 2 Critical Care Research Group, The Prince Charles Hospital, Chermside, Queensland Australia; 3 Australian Centre for Health Services Innovation (AusHSI) and Centre for Healthcare Transformation, School of Public Health and Social Work, Queensland University of Technology (QUT), Brisbane, QLD Australia; 4 Neurointensive Care Unit, Critical Care Department, Sanatorio Pasteur, Chacabuco 675, 4700 Catamarca, Argentina; 5 Department of Critical Care Medicine, Sunnybrook Health Sciences Centre, Toronto, ON Canada; 6 University Department of Anaesthesiology in Pain Medicine, University of Toronto, Toronto, ON Canada; 7 Laboratory of Pulmonary Investigation, Carlos Chagas Filho Institute of Biophysics, Federal University of Rio de Janeiro, Rio de Janeiro, Brazil; 8 IRCCS Ospedale Policlinico San Martino, Genova, Italy; 9 Department of Surgical Sciences and Integrated Diagnostics (DISC), University of Genoa, Genoa, Italy; 10 Division of Neurosciences Critical Care, Department of Neurology, Neurosurgery, Anaesthesiology and Critical Care Medicine and Neurosurgery, Johns Hopkins University School of Medicine, Baltimore, USA

**Keywords:** Stroke, Tracheostomy, Mechanical ventilation, Ischaemic, Haemorrhagic, Critically ill

## Abstract

**Background:**

Stroke patients requiring mechanical ventilation often have a poor prognosis. The optimal timing of tracheostomy and its impact on mortality in stroke patients remains uncertain. We performed a systematic review and meta-analysis of tracheostomy timing and its association with reported all-cause overall mortality. Secondary outcomes were the effect of tracheostomy timing on neurological outcome (modified Rankin Scale, mRS), hospital length of stay (LOS), and intensive care unit (ICU) LOS.

**Methods:**

We searched 5 databases for entries related to acute stroke and tracheostomy from inception to 25 November 2022. We adhered to PRISMA guidance for reporting systematic reviews and meta-analyses. Selected studies included (1) ICU-admitted patients who had stroke (either acute ischaemic stroke, AIS or intracerebral haemorrhage, ICH) and received a tracheostomy (with known timing) during their stay and (2) > 20 tracheotomised. Studies primarily reporting sub-arachnoid haemorrhage (SAH) were excluded. Where this was not possible, adjusted meta-analysis and meta-regression with study-level moderators were performed. Tracheostomy timing was analysed continuously and categorically, where early (< 5 days from initiation of mechanical ventilation to tracheostomy) and late (> 10 days) timing was defined per the protocol of SETPOINT2, the largest and most recent randomised controlled trial on tracheostomy timing in stroke patients.

**Results:**

Thirteen studies involving 17,346 patients (mean age = 59.8 years, female 44%) met the inclusion criteria. ICH, AIS, and SAH comprised 83%, 12%, and 5% of known strokes, respectively. The mean time to tracheostomy was 9.7 days. Overall reported all-cause mortality (adjusted for follow-up) was 15.7%. One in five patients had good neurological outcome (mRS 0–3; median follow-up duration was 180 days). Overall, patients were ventilated for approximately 12 days and had an ICU LOS of 16 days and a hospital LOS of 28 days. A meta-regression analysis using tracheostomy time as a continuous variable showed no statistically significant association between tracheostomy timing and mortality (*β* = − 0.3, 95% CI = − 2.3 to 1.74, *p* = 0.8). Early tracheostomy conferred no mortality benefit when compared to late tracheostomy (7.8% vs. 16.4%, *p* = 0.7). Tracheostomy timing was not associated with secondary outcomes (good neurological outcome, ICU LOS and hospital LOS).

**Conclusions:**

In this meta-analysis of over 17,000 critically ill stroke patients, the timing of tracheostomy was not associated with mortality, neurological outcomes, or ICU/hospital LOS.

*Trial registration*: PROSPERO—CRD42022351732 registered on 17th of August 2022.

**Supplementary Information:**

The online version contains supplementary material available at 10.1186/s13054-023-04417-6.

## Background

Patients with stroke, requiring long-term mechanical ventilation, are at high risk of death and poor neurological outcome [1–3]. Tracheostomy is often considered in these patients, especially when weaning from mechanical ventilation and extubation is difficult or delayed [2, 4]. The rate of tracheostomy is higher in stroke patients compared to the general intensive care unit (ICU) population [5–7].

Tracheostomy reduces airway dead space, decreases oropharyngeal lesions and need for sedatives and increases patient comfort [8, 9]. Tracheostomy in the general ICU population may be delayed by 2 to 3 weeks following intubation, while in stroke patients, tracheostomy is generally considered after 7–14 days [10–12]. Earlier timing [8, 10, 12, 13] has been studied in the stroke population as these patients are more prone to extubation failure due to inability to protect the airway (absent airway reflexes), low Glasgow Coma Score (GCS), and dysphagia due to brainstem involvement [2, 4].

Though flawed, the TracMan randomised controlled trial (RCT) reported that earlier tracheostomy reduced mortality, ICU complications, need for analgesia and sedation, duration of mechanical ventilation and ICU length of stay (LOS) in the general ICU population [14]. In stroke patients, however, results from recent RCTs (SETPOINT and SETPOINT2) have been contentious: SETPOINT (a phase II prospective, randomised, pilot study powered to detect differences in ICU LOS) showed that early tracheostomy (1–3 days following intubation) timing failed to produce a difference in ICU LOS compared to standard tracheostomy timing (7–14 days). However, analysis of secondary aims suggested reduced ICU mortality, sedative use, and 6-month mortality among those with earlier tracheostomy [10]. SETPOINT2 showed no difference in these outcomes between early (< 5 days) and standard tracheostomy timings (> 10 days) [8, 10]. Meanwhile, observational studies showed varying effects of early tracheostomy on outcome [5, 11, 12, 15–22]. Synthesis of such heterogenous evidence is challenging and previous meta-analyses of tracheostomy timing did not adopt a universal definition for early and late tracheostomy. This reliance on study specific, categorical definitions of tracheostomy timing (early vs. late) and lack of consideration for cohort characteristics may obscure benefits. As such, the impact of tracheostomy timing on clinical outcome remains inadequately investigated and lacks consensus [2].

We performed a comprehensive systematic review and meta-analysis to investigate the impact of tracheostomy timing on all-cause mortality in patients with severe stroke. We hypothesise that tracheostomy timing is not associated with all-cause mortality in this population. We further explored the impact of tracheostomy timing on neurological outcome (mRS), ICU/hospital LOS, mechanical ventilation (MV) days as secondary outcomes**.**

## Methods

This systematic review was reported in accordance with the Preferred Reporting Items for Systematic Review and Meta-Analysis [23] (PRISMA check-list, Additional file 1: Item S1). The protocol was registered in PROSPERO 17th of August 2022 (Registration number: CRD4202235173).

### Search strategy and selection criteria

Three reviewers (L.P., C.C., and D.B.) systematically searched PubMed, Google Scholar, EMBASE, Scopus, and the Cochrane trial registry for all published observational and randomised studies as of 25th November 2022. Combinations of the following terms were used to identify all relevant articles: “tracheostomy OR tracheotomy OR trachea AND stroke”. MeSH terms and additional Boolean operators were modified as appropriate. After removal of duplicates, titles, and abstracts of identified studies were independently screened by two authors (L.P. and C.C.). References of these studies were also screened. Full texts of all selected articles were independently screened by two authors (L.P and C.C) for adherence to inclusion criteria: peer-reviewed publications, preprints, and published abstracts were eligible for inclusion. No restrictions were placed on language or geographic region. The selected studies included (1) ICU-admitted patients who had stroke (either acute ischaemic stroke, AIS or intracerebral haemorrhage, ICH) and received a tracheostomy (with known timing) during their stay and (2) > 20 tracheotomised patients. Studies with paediatric population and unknown timing of tracheostomy were also excluded. If selection of studies differed between reviewers, discrepancies were resolved by consensus. If a consensus was not reached, a third reviewer was involved in the process (D.B, S.M.C). In literature, stroke is frequently divided into three categories: AIS, ICH, and sub-arachnoid haemorrhage (SAH) [15]. We attempted to exclude sub-arachnoid nature of haemorrhage [4], and thus studies exclusively investigating SAH were excluded as were those reporting greater than a third of stroke patients with SAH. The reason for such exclusion is that SAH, despite being included in stroke definition by some guidelines, manifests with peculiar and distinctive characteristics; vascular malformation is the primary aetiology and clinical course is complicated by vasospasm, hydrocephalus, Takotsubo cardiomyopathy, or delayed cerebral ischemia [2, 13]. Thus, we aimed to exclude this subgroup of patients to mitigate heterogeneity within patient outcomes.

### Definitions and outcomes

We defined time to tracheostomy as the mean time between initiation of MV and tracheostomy (days). Stroke type was represented by the ratio of acute ischaemic stroke to intracerebral haemorrhage (AIS: ICH) in each study. MV duration was defined as the time (days) between initiation and cessation of MV. Good neurological outcome was defined as mRS between 0 and 3 or Glasgow Outcome Score (GOS) 4–5. Primary analysis treated time to tracheostomy as a continuous variable. When tracheostomy timing was analysed as a categorical variable, early tracheostomy was defined as less than 5 days and late tracheostomy as greater than 10 days as per SETPOINT2 [8]**.** This definition was derived from (1) a pre-study survey of UK Intensive Care Society members and 27 ICUs in the UK performed by the TracMan collaborators found that median time to tracheostomy was 10–11 days after ICU admission [14] with ~ 50% being placed within 5 days of admission [24] (2) timing defined by the most recent RCT in stroke patients (SETPOINT2) [8].

The primary outcome was all-cause mortality in patients with stroke who received tracheostomy. Secondary outcomes were percentage of patients with good neurological outcome (% mRs 0–3), mean mRS, MV duration (days), ICU LOS and hospital LOS (days). Hospital LOS was inclusive of time spent in the ICU.

### Data extraction, synthesis and risk of bias assessment

As per the Population or Problem Intervention or Exposure Comparison Outcome (PICO) approach [25], two reviewers independently extracted data (L.P. and C.C.): study characteristics (study year, duration, stroke type, patient group), patient characteristics (age, gender, GCS on admission), tracheostomy characteristics (mean time to tracheostomy, MV duration) and outcomes (all-cause mortality, percentage of patients with good neurological outcome, mean mRS, ICU LOS, hospital LOS, percentage of patients with ventilatory-associated pneumonia (VAP). When necessary, authors were contacted to obtain missing data. Bias was assessed using the Newcastle Ottawa [26] Quality Assessment Scale (NOS) and RoB-2 tool [27], and statistical methods to assess publication bias were also used as noted below.

### Statistical analysis

All statistical analyses were computed with R studio statistical software. Data were expressed as mean (standard deviation, SD) for continuous variables and number (percentages, %) for categorical variables. Transformations from median (interquartile range, IQR) to estimated mean [28] (SD) [29–31] for use in meta-analysis were performed as described in Additional file 1: Item S2.

The preceding analysis was developed upon consultation with authors (L.P, C.C, D.B, S.M.C) and a statistician (N.W). Meta-analysis was conducted to obtain pooled estimates for tracheostomy time, all-cause mortality, mean mRS, ICU and hospital LOS, MV days, and VAP. Pooled estimates were obtained using random-effects models, assuming an identity link for continuous variables and a logit link (GLM) for binary variables. Using the 'predict’ function, pooled and induvial study estimates were adjusted for follow-up time or proportion of patients with SAH. We used random-effects models accounted for expected between-study heterogeneity. Between-study heterogeneity was assessed using the Cochrane *Q* test, tau [2], and the Higgins *I*^2^ statistic. Confidence intervals (CIs) for binary outcomes were calculated using Wilson scores with between-study variation estimated using the Hartung–Knapp–Sidik–Jonkman method [32].

Using the “metafor" package [33], mixed-effects meta-regression models were used to assess evidence of associations between mean time to tracheostomy (independent variable) and outcome (dependent variable). Where possible, GCS on admission, ratio of ischaemic stroke to haemorrhagic stroke, study year and study duration were included as moderator variables. Models were reported/interpreted using the principle of parsimony and acknowledging the number of observations and potential overfitting. Statistical significance was set at *p* < 0.05.

We further explored the ability of a priori defined, clinically relevant, study-level variables (mean time to tracheostomy, GCS, stroke type) to model outcome data. Using the “MuMIn” package in R for multi-model inference [34, 35], corrected Akaike’s information criterion (AICc) was computed for all combinations of the above variables. The results from the model selection were used to assess variable importance for explaining variation in outcomes (Additional file 1: Item S2).

## Results

**Fig. 1:**
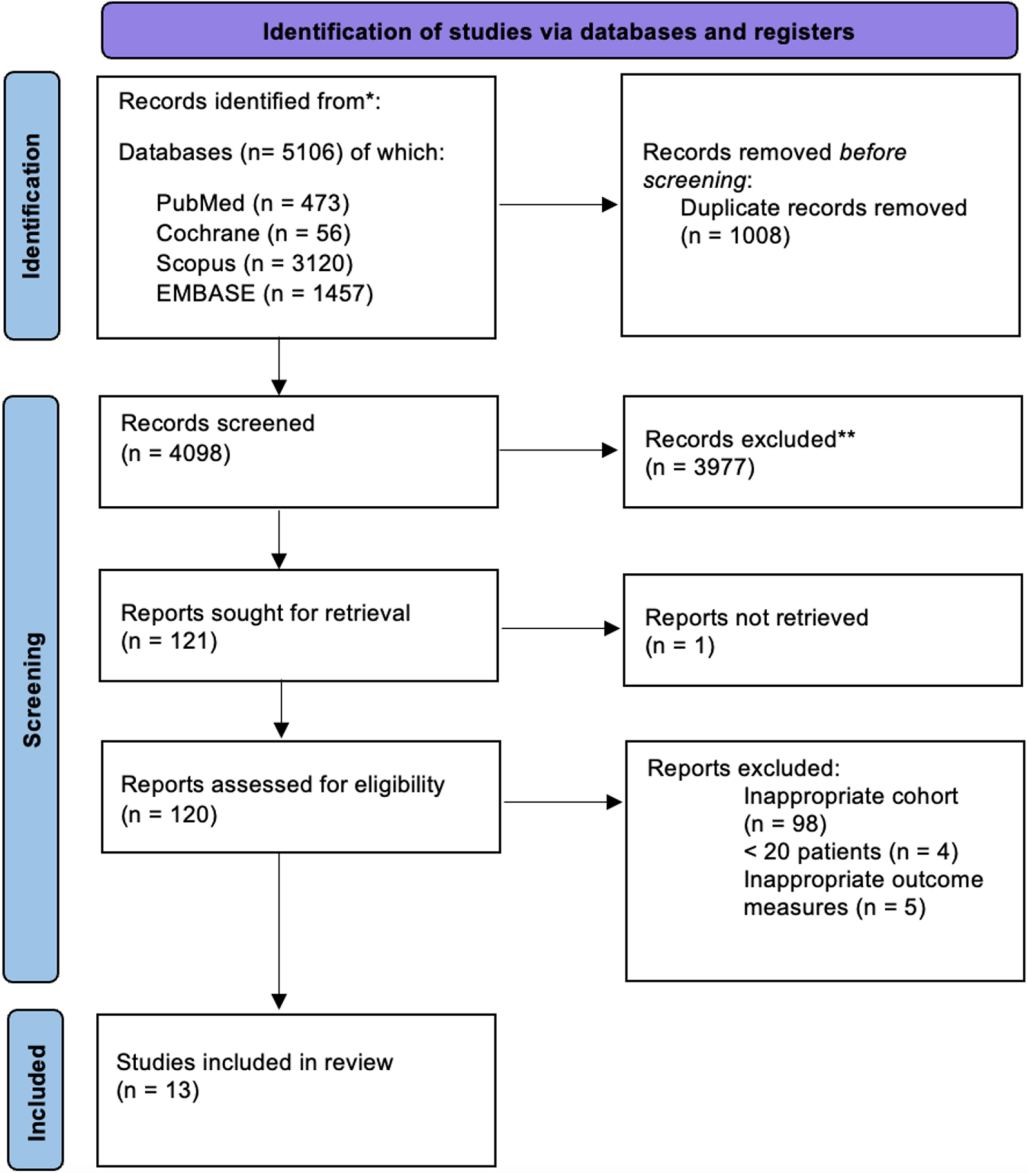
PRISMA flow chart for study selection and inclusion

The initial search yielded 4,098 individual studies of which 13 studies were suitable for analysis: ten retrospective cohort studies [5, 11–13, 15–22, 36], two RCTs [8, 10] and one prospective cohort study [20]. Table 1 summarises characteristics of included studies. The search and selection strategy are represented in Fig. 1.

**Table 1:** Summary of included study characteristics

Author	Year	Study type	Setting	Overall bias (NOS)	Total number of patients	Mean tracheostomy time (days, SD)
Alsherbini et al	2019	Retrospective Cohort Study	Single-centre	8	140	–
Bosel et al	2013	Randomised controlled trial	Single-centre	–*	60	6.62 (0.35)
Bosel et al	2022	Randomised controlled trial	Multi-centre	–*	380	7.2 (0.43)
Catalino et al	2018	Retrospective cohort study	Single-Centre	9	48	–
Chen et al	2019	Retrospective cohort study	Single-centre	9	425	4.25 (2.59)
Hallan et al	2022	Retrospective Cohort Study	Multi-centre	8	2420	12.58
Lee et al	2015	Retrospective cohort study	Single-centre	8	95	–
Maier et al	2021	Retrospective cohort study	Single-centre	8	40	16
Pelosi et al	2011	Retrospective cohort study	Multi-centre	8	362	9.19 (3.68)
Rabinstein et al	2004	Retrospective cohort study	Single-centre	8	97	14.77 (53.1)
Schneider et al	2017	Prospective cohort study	Single-centre	8	53	11.33 (5.27)
Shen et al	2022	Retrospective cohort study	Single-centre	8	61	6.98 (6.12)
Villwock et al	2014	Retrospective cohort study	Multi-centre	8	13,165	12.00 (4.17)

*NOS* Newcastle–Ottawa Scale, *SD* standard deviation

*As the marked studies are RCTs the NOS system cannot be used to assess study bias

### Patient characteristics

In total, 17,346 stroke patients (9,810 males [56.6%], 7,536 females [43.4%]) were included with mean age of 59.8 years (95% CI = 56.6–62.9). Where stroke type was known, 478 patients (11.5%) had AIS, 3,458 (83.3%) had ICH, and 216 (5.2%) had SAH. Stroke type was not classified in two studies (13,194 patients, Additional file 1: Item S3) [19, 22]. The mean tracheostomy time was 9.7 days (95% CI = 7.3–12.0, Additional file 1: Item S4). The frequency of VAP [5, 11, 21, 22] and of tracheostomy -related complications [10, 20] are presented in Additional file 1: Item S5.

### Quality and risk of bias assessment

Additional file 1: Item S6 provides an assessment of methodological quality (Newcastle–Ottawa Scale and Cochrane risk-of-bias tool for randomized trials) for included studies. All 13 studies were deemed to be good quality (average NOS score 8.2 ± 0.4, funnel plots and Egger’s test results for funnel plot asymmetry are provided in Additional file 1: Item S7).

### Mortality and tracheostomy complications

**Fig. 2:**
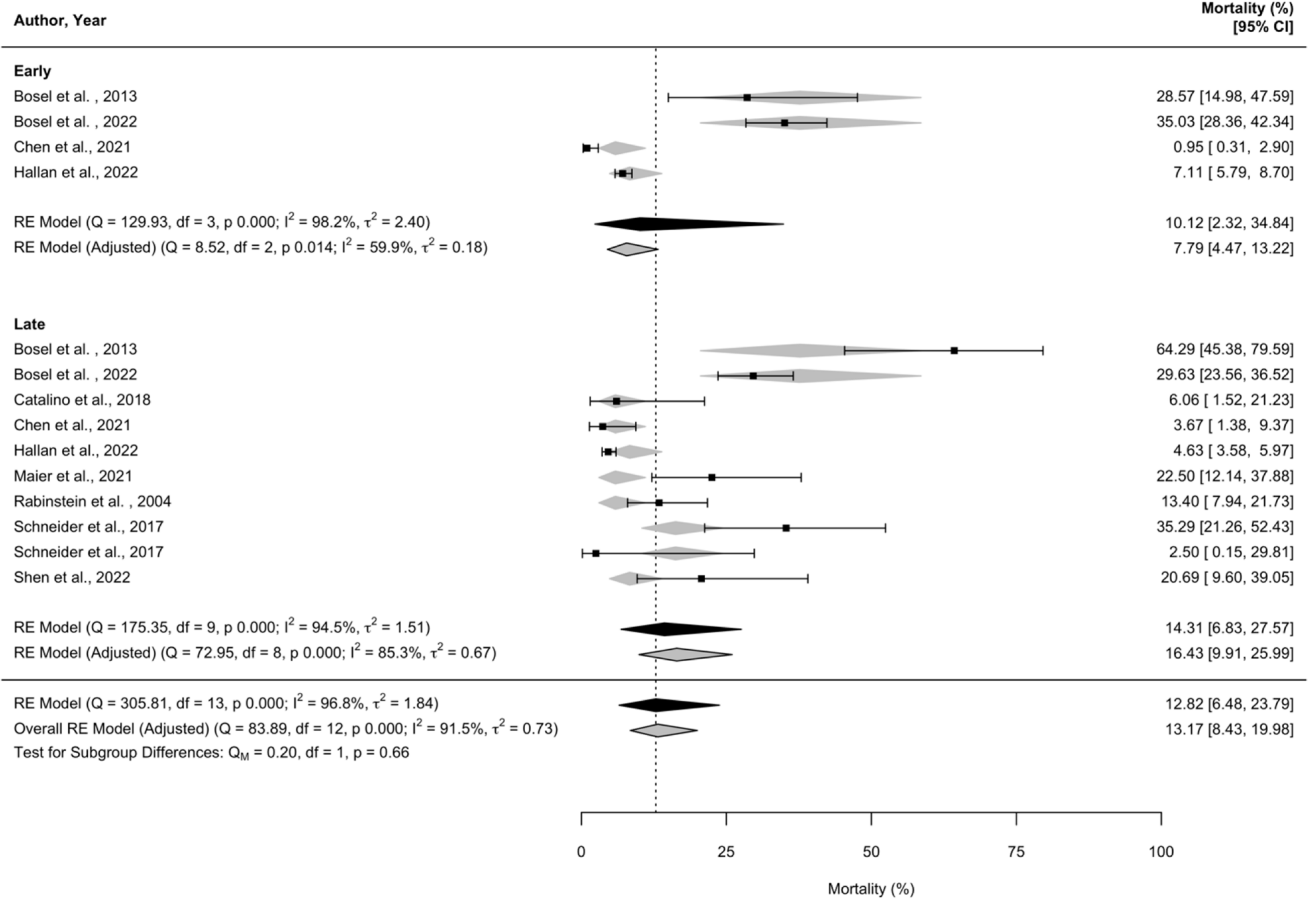
Forest plot of all-cause mortality by subgroup (early tracheostomy, < 5 days and late tracheostomy > 10 days). Unadjusted estimates (black) and estimates adjusted for follow-up time (grey) are shown

Unadjusted, overall mortality was 15.8% (95% CI = 9.4–25.4, *I*^2^ = 98.4%, Additional file 1: ItemS8A). When adjusted for follow-up time, overall mortality was similar (15.7%, Additional file 1: Item S8B). ICU mortality was 26.3% (95% CI = 16.0–40.0, *I*^2^ = 91.1%, Additional file 1: Item S9). Upon classifying tracheostomy timing as early (< 5 days) and late (> 10 days), there was no difference in all-cause mortality between the groups (*p* = 0.7, Fig. 2).

**Fig. 3:**
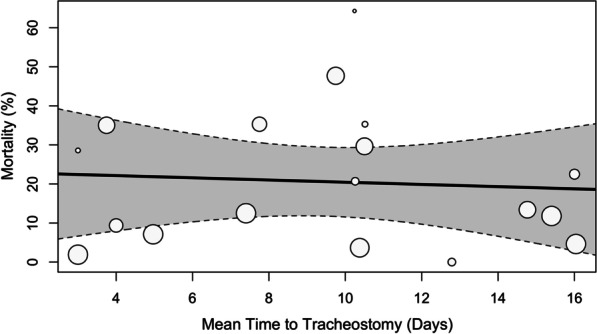
Meta-regression of mortality (%) versus mean time to tracheostomy (days) without moderators; estimate = − 0.28, 95% CI = − 2.30–1.74, *p* = 0.77. Grey envelope indicates 95% CI

The meta-regression did not show a statistically significant association between the mean time to tracheostomy and mortality (estimate = − 0.3, 95% CI = −2.3–1.7, *p* = 0.8, Fig. 3). Meta-regression with moderators, study year, follow-up, stoke type (AIS vs. ICH) and GCS on admission, still had significant residual heterogeneity (R^2^ = 0.0%).

**Fig. 4:**
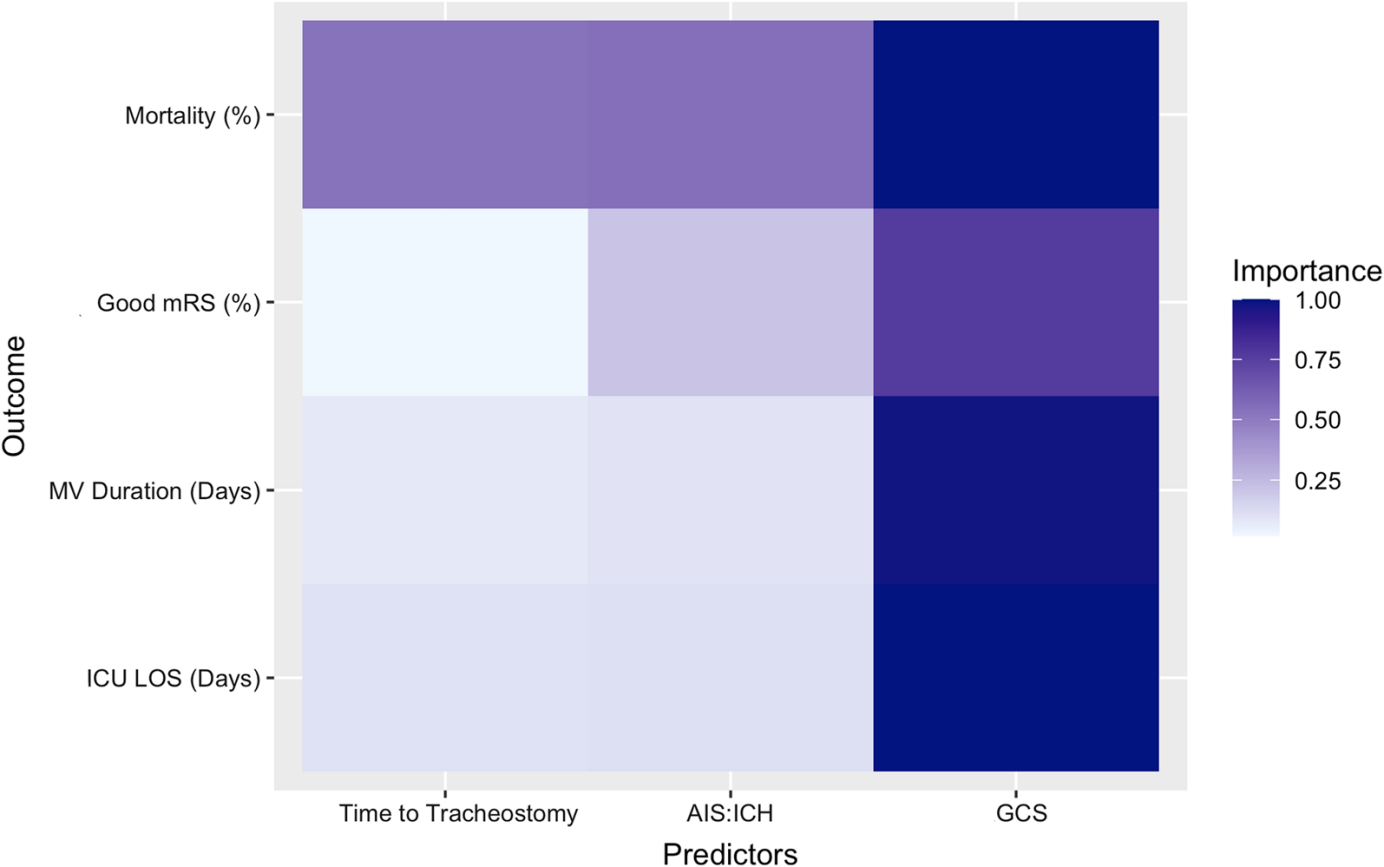
Heatmap of relative predictor importance. Predictor importance gives the averaged Akaike weight of each predictor (time to tracheostomy, stroke type [AIS: ICH] and GCS on admission [GCS]) averaged across all models in which it appears (i.e. the variable that appears in the most models with highest weights has importance closer to 1.0—see Additional file 1: Item S2)

Among the subset of variables explored using multi-model meta-regression, the combination of GCS on admission and AIS: ICH returned a better goodness of fit compared with mean time to tracheostomy alone (Additional file 1: Item S10, Fig. 4).

### Neurological outcome

Overall, around a fifth of patients had good neurological outcome (mRS 0–3, median length of follow-up 180 days: IQR = 135 to 365 days, Additional file 1: Item S11A). Adjusting for follow-up did not affect the pooled estimate (Additional file 1: Item S11A). The proportion of patients with good neurological outcome did not vary with time to tracheostomy (estimate = − 0.2 95% CI = − 2.6–2.2, *p* = 0.8. Additional file 1: Item S12). Meta-regression with moderators (study year, AIS vs. ICH and GCS on admission) did not explain any further heterogeneity (*R*^2^ = 0.0%). Further, time to tracheostomy demonstrated no statistically significant association with mean mRS score (Additional file 1: Item S13). AIS: ICH explained more variation in good neurological outcome compared with mean time to tracheostomy (Fig. 4 and Additional file 1: Item S10).

### Hospitalisation characteristics: length of stay and MV duration

Overall hospital LOS was 27.7 days (95% CI = 21.2 to 34.3, *I*^2^ = 94.9%, Additional file 1: Item S11B). Time to tracheostomy was not associated with hospital LOS (Additional file 1: ItemS12). Analysis of mechanical ventilation duration showed no association with time to tracheostomy (Additional file 1: Item S12, Item S14).

Overall ICU LOS was 16.4 days (95% CI = 12.4 to 20.4, *I*^2^ = 94.3%, Additional file 1: Item S11C). ICU LOS was not different between early (< 5 days) and late (> 10 days) groups (Additional file 1: Item S15). Meta-regression indicated that mean time to tracheostomy was not associated with ICU LOS (Additional file 1: Item S12). GCS on admission explained the most variation in ICU LOS across studies (Additional file 1: Item S10).

## Discussion

Our systematic review and meta-analysis are the largest to date and comprehensively describes the effect of tracheostomy timing on patient outcome (all-cause mortality, neurological outcome, hospital/ICU stay and MV duration). Our analysis of over 17,000 critically ill stroke patients demonstrated no associations between time to tracheostomy and the clinical outcomes investigated. Further, our findings suggested that GCS on admission and stroke type are better predictors of outcome than time to tracheostomy. Evidently, clinical outcomes in critically ill stroke patients depend on a multifactorial array of variables, only one of which is the timing of tracheostomy [3].

Several studies have investigated the effect of tracheostomy timing in the ICU [37] yielding unclear and, at times, contradictory results [2]. Apart from one RCT sufficiently powered and structured (SETPOINT2), studies on this topic are mainly retrospective and carry with them inherent methodological limitations. Previous meta-analyses utilised study specific definitions of early and late tracheotomy with no standardised definition [37, 38]. This lack of standardisation again limits the comparability of findings across studies. Significant heterogeneity brought about by patient, disease, and institutional factors also contributed to discrepancies in the observable benefit of early tracheostomy. Given this, our analysis provides considerable insight into the effect of tracheostomy timing on patient outcome by (1) applying a global definition (SETPOINT2) for early and late tracheostomy as well as analysing tracheostomy timing as a continuous variable (2) restricting our study population to AIS or ICH.

### Implications of our findings

We demonstrated that tracheostomy timing was not associated with all-cause mortality, using a clinically relevant [2, 8, 14, 24] categorical definition of early (< 5 days) and late (> 10 days) tracheostomy and as a continuous variable in days. Auxiliary analysis showed that GCS on admission was the best predictor of mortality, perhaps serving as a proxy for stroke severity in mixed stroke cohorts (especially given National Institutes of Health Stroke Score and ICH score was poorly reported in the included studies) [2, 4]. Despite the inclusion of study-level moderators in our models, ample heterogeneity remained, indicating significant variability between the included studies. Overall, our study suggests that the timing of tracheostomy does not change the course of their ICU illness [3, 4, 9, 37].

Two randomised trials in critically ill stroke patients were conducted: the single-centre pilot trial SETPOINT and the multi-centre trial SETPOINT2. SETPOINT yielded neutral results for the primary endpoint (ICU LOS) but suggested association between earlier tracheostomy and secondary outcomes [8, 10]. SETPOINT2, which had sufficient power to examine neurological outcomes as its primary endpoint, did not find a difference in survival without severe disability (at 6 months), between the early and late tracheostomy group [8]. We showed that tracheostomy timing was neither associated with the proportion of patients with good neurological outcome (mRS 0–3) nor mean mRS score (Additional file 1: Item S13). The physiological rationale to support the hypothesis that earlier tracheostomy contributes to faster neurological recovery post-stroke derives from the benefits of tracheostomy over endotracheal tube for long-term MV. Specifically earlier tracheostomy may decrease work of breathing [39, 40] and therefore potentially allows for greater brain energy consumption during recovery. Additionally, tracheostomy may benefit intracranial pressure control [41, 42], reduces need for sedatives and may facilitate earlier weaning, mobilisation, and transfer to rehabilitation [10, 22, 37]. However, it is unlikely these benefits manifest better long-term neurological outcome. Further, the additional moderators included in our models (study year, GCS score, stroke type (AIS:ICH) and time to tracheostomy) may initially affect an individual’s clinical course but their effect on neurological recovery is unlikely to be appreciable at the study level and may be outweighed by longer weaning duration [8].

The effect of tracheostomy timing on ICU and hospital LOS is highly variable. In the general ICU population, the TracMan (2013) RCT found no difference in ICU LOS between early (< 4 days) and late groups (> 10 days) [14]. Similar findings were obtained by SETPOINT when the same endpoint (ICU LOS) was studied in the ICU stroke population [10]. In accordance, we report no association between tracheostomy timing (continuous variable) and LOS. Despite the paucity of RCT evidence in the stroke population, synthesis of current data showed early versus late (< 5 day and > 10-day, respectively) cut-offs had no impact on ICU LOS. Heterogeneity persisted despite the addition of moderators describing disease severity. Factors associated with ICU course (eligibility for fibrinolysis, surgical intervention, tracheostomy complications, weaning duration) may be more important determinants of ICU LOS [3]. The relationship between tracheostomy timing and mechanical ventilation duration is discussed in detail in Additional file 1: Item S14. Briefly, we report that severe neurological injury, prolongation of severe respiratory failure, difficulty weaning and inability to protect-airway may lengthen MV duration and LOS regardless of earlier tracheostomy [2, 8]. Indeed, time to tracheostomy was outperformed as a predictor of ICU LOS and MV duration by traditional indicators of disease severity (GCS on admission, stroke type). Our population was primarily ICH. As such our findings are perhaps most relevant to this population and further study is needed in patients with AIS. Patients with SAH were excluded and are likely to have vastly different hospital trajectories; investigation into the effect of tracheostomy timing in this sub-population is also warranted [43, 44].

### Limitations

It is important to acknowledge key limitations of our findings. First, a small number of patients with known SAH (n = 216, 5.2%) were included in cohorts with primarily AIS or ICH. Second, our study consisted of one sufficiently powered RCT combined with multiple smaller observational studies. Of these, one study constituted ~ 75% of patients. Additional file 1: Item S2 provides a detailed analysis of this effect and justification of the findings presented. Similarly, despite attempts to classify strokes by aetiology, a substantial proportion of strokes were unspecified (76%), albeit in only two studies. Third, not all patients in Bosel et al., 2022 received a tracheostomy. Sensitivity analyses, adjusting for the proportion of SAH (Additional file 1: Item S8C) and removing problematic studies (Additional file 1: Item S16) did not alter our findings. Fourth, missing parameters impacted our study: stroke severity was inferred by GCS on admission only (especially given that NIHSS or ICH score were poorly reported, Additional file 1: Item S17)). Interventions such as fibrinolytics, decompressive craniectomy/craniotomy, duration of sedation and weaning protocols were infrequently reported, as were complications of stroke, tracheostomy, and follow-up time. Many of the above parameters are reported to play an important role in predicting tracheostomy needs [15, 45]. Indeed, studies should attempt to expand and validate predictors of tracheostomy needs in stroke patients. Current tools to estimate tracheostomy need, include the TRACH score for patients with supratentorial spontaneous ICH [46], the SETscore in ICU patients with severe stroke [15, 47], and the RAISE score in SAH [48]. Finally, we emphasise that our findings are largely based on meta-analysis of observational studies; therefore, inferring causal relationships is not possible. Indeed, there is only one large RCT in this population, our analysis derives primarily from retrospective data. Regardless, our analysis employed robust methods to synthesise current evidence and investigate the relationship between the mean time to tracheostomy and clinical outcomes. We highlighted moderators that optimally modelled the data, without implying causation. In doing so, we derived clinically meaningful, novel results around the impact of tracheostomy timing on patient outcome. Based on our analysis, there is no evidence to support an effect of tracheostomy timing on the outcomes of critically ill stroke patients, despite the absence of randomised, prospective data; nevertheless, further research is necessary to substantiate these findings in patients with SAH and confirm our findings in larger populations of AIS patients.

## Conclusions

The present meta-analysis included over 17,000 critically ill stroke patients and showed that timing of tracheostomy was not associated with mortality, neurological outcome, ICU/hospital LOS or MV duration. We recommend clinical decisions around tracheostomy be based on patient characteristics, neurological status and prognosis, risk–benefit ratio, patient comfort and requests of patients and caregivers.

## Supplementary Information

**Additional file 1. Item S1:** PRISMA Checklist; **Item S2:** Additional Methods (Statistical Analysis) - Summary of Sensitivity Analysis: Villwock et al., (2014); **Item S3:** Outcomes reported (by study); **Item S4:** Mean time to Tracheostomy (forest-plot); **Item S5:** Unadjusted (A) and follow up adjusted overall mortality (B); **Item S6:** NOS study quality and bias assessment; **Item S7:** Funnel plots & test of plot asymmetry; **Item S8:** Ventilator Associated Pneumonia (forest-plot); **Item S9:** ICU mortality overall estimate; **Item S10:** Sensitivity analysis (Mortality): Early vs. Late tracheostomy (subgroup); **Item S11:** Multimodel Interference outputs with Information Criteria (AICc) and Weights (A-E); **Item S12:** Proportion of good neurological outcome (mRS 0-3, %), Mechanical Ventilation Duration, Hospital Length of Stay, ICU-Length of stay (forest-plot, A-D); **Item S13:** Meta-regression outputs; **Item S14:** Additional Results (Mean mRS score); **Item S15:** Additional Results and Discussion (Mean MV Duration); **Item S16:** SETPOINT-2 threshold interaction term outputs (Mortality and ICU-LOS); **Item S17:** Test of correlation between moderator variables.

## Data Availability

See Additional file in addition to Results. Additional data will be available to corresponding author under reasonable request.
