# Supplementary material for: Tracheostomy timing and outcome in critically ill patients with stroke: a meta-analysis and meta-regression
Source: Crit Care. 2023 Apr 1;27:132. doi: 10.1186/s13054-023-04417-6 (PMC10068163; doi:10.1186/s13054-023-04417-6)
Supplement: Supplementary file 1 — Additional file 1. Item S1: PRISMA Checklist; Item S2: Additional Methods (Statistical Analysis) - Summary of Sensitivity Analysis: Villwock et al., (2014); Item S3: Outcomes reported (by study); Item S4: Mean time to Tracheostomy (forest-plot); Item S5: Unadjusted (A) and follow up adjusted overall mortality (B); Item S6: NOS study quality and bias assessment; Item S7: Funnel plots & test of plot asymmetry; Item S8: Ventilator Associated Pneumonia (forest-plot); Item S9: ICU mortality overall estimate; Item S10: Sensitivity analysis (Mortality): Early vs. Late tracheostomy (subgroup); Item S11: Multimodel Interference outputs with Information Criteria (AICc) and Weights (A-E); Item S12: Proportion of good neurological outcome (mRS 0-3, %), Mechanical Ventilation Duration, Hospital Length of Stay, ICU-Length of stay (forest-plot, A-D); Item S13: Meta-regression outputs; Item S14: Additional Results (Mean mRS score); Item S15: Additional Results and Discussion (Mean MV Duration); Item S16: SETPOINT-2 threshold interaction term outputs (Mortality and ICU-LOS); Item S17: Test of correlation between moderator variables. [file 13054_2023_4417_MOESM1_ESM.docx]

**ADDITIONAL FILE 1**

**ADDITIONAL FILE 1 – ITEM S1:** PRISMA Checklist

**ADDITIONAL FILE 1 – ITEM S2:** Additional Methods (Statistical Analysis) and Summary of Sensitivity Analysis Findings

**ADDITIONAL FILE 1 – ITEM S3:** Outcomes reported (by study)

**ADDITIONAL FILE 1 – ITEM S4:** Mean time to Tracheostomy (forest-plot)

**ADDITIONAL FILE 1 – ITEM S5:** Ventilator Associated Pneumonia (forest-plot)

**ADDITIONAL FILE 1 – ITEM S6:** NOS study quality and bias assessment and Cochrane Risk of Bias Assessment Tool 2 (RoB 2)

**ADDITIONAL FILE 1 – ITEM S7:** Funnel plots & test of plot asymmetry

**ADDITIONAL FILE 1 – ITEM S8:** Unadjusted (A) and follow up adjusted overall mortality (B)

**ADDITIONAL FILE 1 – ITEM S9:** ICU mortality overall estimate

**ADDITIONAL FILE 1 – ITEM S10:** Multimodel Interference outputs with Information Criteria (AICc) and Weights (A-E)

**ADDITIONAL FILE 1 – ITEM S11:** Proportion of good neurological outcome (mRS 0-3, %), Mechanical Ventilation Duration, Hospital Length of Stay, ICU-Length of stay (forest-plot, A-D)

**ADDITIONAL FILE 1 – ITEM S12:** Meta-regression outputs

**ADDITIONAL FILE 1 – ITEM S13:** Additional Results (Mean mRS score)

**ADDITIONAL FILE 1 – ITEM S14:** Additional Results and Discussion (Mean MV Duration)

**ADDITIONAL FILE 1 – ITEM S15:** SETPOINT-2 threshold interaction term outputs (Mortality and ICU-LOS)

**ADDITIONAL FILE 1 – ITEM S16:** Sensitivity analysis (Mortality): Early vs. Late tracheostomy (subgroup)

**ADDITIONAL FILE 1 – ITEM S17:** Test of correlation between moderator variables

**ADDITIONAL FILE 1 – ITEM S1:** PRISMA Checklist

| **Section and Topic** | **Item #** | **Checklist item** | **Location where item is reported** |
| --- | --- | --- | --- |
| **TITLE** | | |  |
| Title | 1 | Identify the report as a systematic review. | Pg. 1 |
| **1ABSTRACT** | | |  |
| Abstract | 2 | See the PRISMA 2020 for Abstracts checklist. | Pg. 3 |
| **INTRODUCTION** | | |  |
| Rationale | 3 | Describe the rationale for the review in the context of existing knowledge. | Pg. 5-6 |
| Objectives | 4 | Provide an explicit statement of the objective(s) or question(s) the review addresses. | Pg. 5-6 |
| **METHODS** | | |  |
| Eligibility criteria | 5 | Specify the inclusion and exclusion criteria for the review and how studies were grouped for the syntheses. | Pg. 6-7 |
| Information sources | 6 | Specify all databases, registers, websites, organisations, reference lists and other sources searched or consulted to identify studies. Specify the date when each source was last searched or consulted. | Pg. 6 |
| Search strategy | 7 | Present the full search strategies for all databases, registers and websites, including any filters and limits used. | Pg. 6-7 |
| Selection process | 8 | Specify the methods used to decide whether a study met the inclusion criteria of the review, including how many reviewers screened each record and each report retrieved, whether they worked independently, and if applicable, details of automation tools used in the process. | Pg. 6-7 |
| Data collection process | 9 | Specify the methods used to collect data from reports, including how many reviewers collected data from each report, whether they worked independently, any processes for obtaining or confirming data from study investigators, and if applicable, details of automation tools used in the process. | Pg. 6-7 |
| Data items | 10a | List and define all outcomes for which data were sought. Specify whether all results that were compatible with each outcome domain in each study were sought (e.g. for all measures, time points, analyses), and if not, the methods used to decide which results to collect. | Pg. 7-8 |
|  | 10b | List and define all other variables for which data were sought (e.g. participant and intervention characteristics, funding sources). Describe any assumptions made about any missing or unclear information. | Pg. 7-8 |
| Study risk of bias assessment | 11 | Specify the methods used to assess risk of bias in the included studies, including details of the tool(s) used, how many reviewers assessed each study and whether they worked independently, and if applicable, details of automation tools used in the process. | Pg. 8 |
| Effect measures | 12 | Specify for each outcome the effect measure(s) (e.g. risk ratio, mean difference) used in the synthesis or presentation of results. | Pg. 7-8 |
| Synthesis methods | 13a | Describe the processes used to decide which studies were eligible for each synthesis (e.g. tabulating the study intervention characteristics and comparing against the planned groups for each synthesis (item #5)). | Pg. 7-8 |
|  | 13b | Describe any methods required to prepare the data for presentation or synthesis, such as handling of missing summary statistics, or data conversions. | Pg. 8-9 |
|  | 13c | Describe any methods used to tabulate or visually display results of individual studies and syntheses. | Pg. 8-9 |
|  | 13d | Describe any methods used to synthesize results and provide a rationale for the choice(s). If meta-analysis was performed, describe the model(s), method(s) to identify the presence and extent of statistical heterogeneity, and software package(s) used. | Pg. 8-9 |
|  | 13e | Describe any methods used to explore possible causes of heterogeneity among study results (e.g. subgroup analysis, meta-regression). | Pg. 8-9 |
|  | 13f | Describe any sensitivity analyses conducted to assess robustness of the synthesized results. | Pg. 8-9 |
| Reporting bias assessment | 14 | Describe any methods used to assess risk of bias due to missing results in a synthesis (arising from reporting biases). | Pg. 8-9 |
| Certainty assessment | 15 | Describe any methods used to assess certainty (or confidence) in the body of evidence for an outcome. | Pg. 8-9 |
| **RESULTS** | | |  |
| Study selection | 16a | Describe the results of the search and selection process, from the number of records identified in the search to the number of studies included in the review, ideally using a flow diagram. | Pg. 9-10 |
|  | 16b | Cite studies that might appear to meet the inclusion criteria, but which were excluded, and explain why they were excluded. | Figure 1 |
| Study characteristics | 17 | Cite each included study and present its characteristics. | Table 1 |
| Risk of bias in studies | 18 | Present assessments of risk of bias for each included study. | Supplemental Material 4. Supplemental Table 3, 4. |
| Results of individual studies | 19 | For all outcomes, present, for each study: (a) summary statistics for each group (where appropriate) and (b) an effect estimate and its precision (e.g. confidence/credible interval), ideally using structured tables or plots. | Pg. 9-12 |
| Results of syntheses | 20a | For each synthesis, briefly summarise the characteristics and risk of bias among contributing studies. | Pg. 10 |
|  | 20b | Present results of all statistical syntheses conducted. If meta-analysis was done, present for each the summary estimate and its precision (e.g. confidence/credible interval) and measures of statistical heterogeneity. If comparing groups, describe the direction of the effect. | Pg. 9-12 |
|  | 20c | Present results of all investigations of possible causes of heterogeneity among study results. | Pg. 9-12 |
|  | 20d | Present results of all sensitivity analyses conducted to assess the robustness of the synthesized results. | Pg. 12-15 |
| Reporting biases | 21 | Present assessments of risk of bias due to missing results (arising from reporting biases) for each synthesis assessed. | Pg. 13 |
| Certainty of evidence | 22 | Present assessments of certainty (or confidence) in the body of evidence for each outcome assessed. | Pg. 9-12 |
| **DISCUSSION** | | |  |
| Discussion | 23a | Provide a general interpretation of the results in the context of other evidence. | Pg. 12-15 |
|  | 23b | Discuss any limitations of the evidence included in the review. | Pg. 13 |
|  | 23c | Discuss any limitations of the review processes used. | Pg. 13 |
|  | 23d | Discuss implications of the results for practice, policy, and future research. | Pg. 12-15 |
| **OTHER INFORMATION** | | |  |
| Registration and protocol | 24a | Provide registration information for the review, including register name and registration number, or state that the review was not registered. | Pg. 6 |
|  | 24b | Indicate where the review protocol can be accessed, or state that a protocol was not prepared. | Pg. 6 |
|  | 24c | Describe and explain any amendments to information provided at registration or in the protocol. | N/A |
| Support | 25 | Describe sources of financial or non-financial support for the review, and the role of the funders or sponsors in the review. | Pg. 1 |
| Competing interests | 26 | Declare any competing interests of review authors. | Pg. 1 |
| Availability of data, code and other materials | 27 | Report which of the following are publicly available and where they can be found: template data collection forms; data extracted from included studies; data used for all analyses; analytic code; any other materials used in the review. | Table 1 |

**ADDITIONAL FILE 1 – ITEM S2:** Additional Methods (Statistical Analysis) and Summary of Sensitivity Analysis Findings

**Additional Methods (Statistical Analysis)**

***Transformation of Variables (Methods)***

Transformations from median (interquartile range) to estimated mean (SD)^33,34^ were performed as follows^32^:

$$Estimated mean \left( eMean \right)=\frac{l+2m+u}{4}+\frac{l+2m+u}{4ss}$$

$$Estimated SD \left( eSD \right)=\frac{1}{12}\left( \frac{\left( l-2m+u \right)^{2}}{4}+\left( u-l \right)^{2} \right)$$

$$\mathrm{where}m=median, l=lower, u=upper, ss=sample size$$

Where zero standard deviation was reported, the Furukawa method for standard deviation imputation was used.

During meta-analysis, generalised linear model (GLMM) was used and therefore proportions were logit transformed as required.

***Model Selection and Averaging Methods***

Model parameter and prediction averaging was based on model weights derived from AICc. The top 5 models were generated and interpreted with awareness of potential-overfitting and parsimony. Predictor importance was numerically determined by the average Akaike weight of each predictor averaged across models in which it appears (i.e., a variable that appears in all models with high weights will have importance near 1.0).**See** [**https://www.metafor-project.org/doku.php/tips:model_selection_with_glmulti_and_mumin**](https://www.metafor-project.org/doku.php/tips:model_selection_with_glmulti_and_mumin) for further clarification.

***Sensitivity Analysis (Methods)***

Leave-one-out meta-analysis was performed to determine the effect of each study on the overall estimate (mortality, primary aim). Cohort sizes for other estimates were more uniformly distributed and so further analysis of secondary aims was not deemed relevant. The “rma.glmm” function was used in R and due to incompatibility with the native “leave1out” function, leave-one-out meta-analysis was manually encoded. The results are displayed below **(Figure 1).**

Cooks’ distances for the primary meta-regression model (mortality vs. mean time to tracheostomy) were calculated to evaluate the influence of individual studies on the model. Generalised linear model (“glmer”) with random effects was preformed to replicate meta-regression. Then, cook’s distances were computed with the “cooks.distance()” command. The output is displayed below **(Figure 2).**

**Summary of Sensitivity Analysis Findings**

Our systematic review with meta-analysis that combines many small retrospective analyses, one high quality RCT and one registry study that adds 13,000 patients. This population (Villwock et al., 2014) alone constitutes ~75% of our total study population, derived from 13 studies.

The key methodological measures taken to mitigate the effect of this large population are (1) the use of random-effects meta-analysis and meta-regression (2) sensitivity analysis presented below.

***Random Effects Meta-Analysis***

Unlike fixed effects, random-effects meta-analysis true effect size varies across studies and that the observed effect sizes in each study are a combination of the true effect size and random error. In a study of large sample size, the estimated effect size may be more precise and have less sampling variability.

In a fixed effects meta-analysis, this increased precision would lead to a greater weight being assigned to that study, potentially influencing the result. In contrast, in a random effects meta-analysis, the between-study variability parameter (tau-squared) accounts for the heterogeneity among the true effect sizes across studies, and thus the influence of any single study is more limited. This is also true for meta-regressions utilising random effects.

***Further Sensitivity Analyses***

Leave-one-out Meta-Analysis

To further clarify the effect of Villwock et al., on our results, leave-one out meta-analysis **(Figure 1)** was performed for the primary aim (mortality) and demonstrates that the overall estimate (15.82%, unadjusted overall mortality) was not affected substantially by exclusion of Villwock et al. (15.93%). Note: Villwock et al., was not included in our analysis of tracheostomy timing as a categorical variable (see **Figure 2 [manuscript]**, as the definition used as <5 days [early] and >10 days [late] did not include the timing reported by Villwock et al; 7.4 and 15.4 days respectively). See also above for clarification **ITEM S2**.

Cook’s distances for Meta-Regression Model

Lastly, to determine the effect of effect size on our model, Cook's distance was computed. This is a measure of the influence of each observation on the regression and is based on the change in the model when each observation is deleted. Cooks distance >1 is considered highly influential. **Figure 2** shows the cooks distance plot for our primary meta-regression (overall mortality versus mean time to tracheostomy). See also for clarification Statistical Methods Continued (above).

**Figure 1:** Results of Leave-One-Out-Meta-Analysis (Mortality). The overall effect estimate (excluding the corresponding study) is given by the black dot.


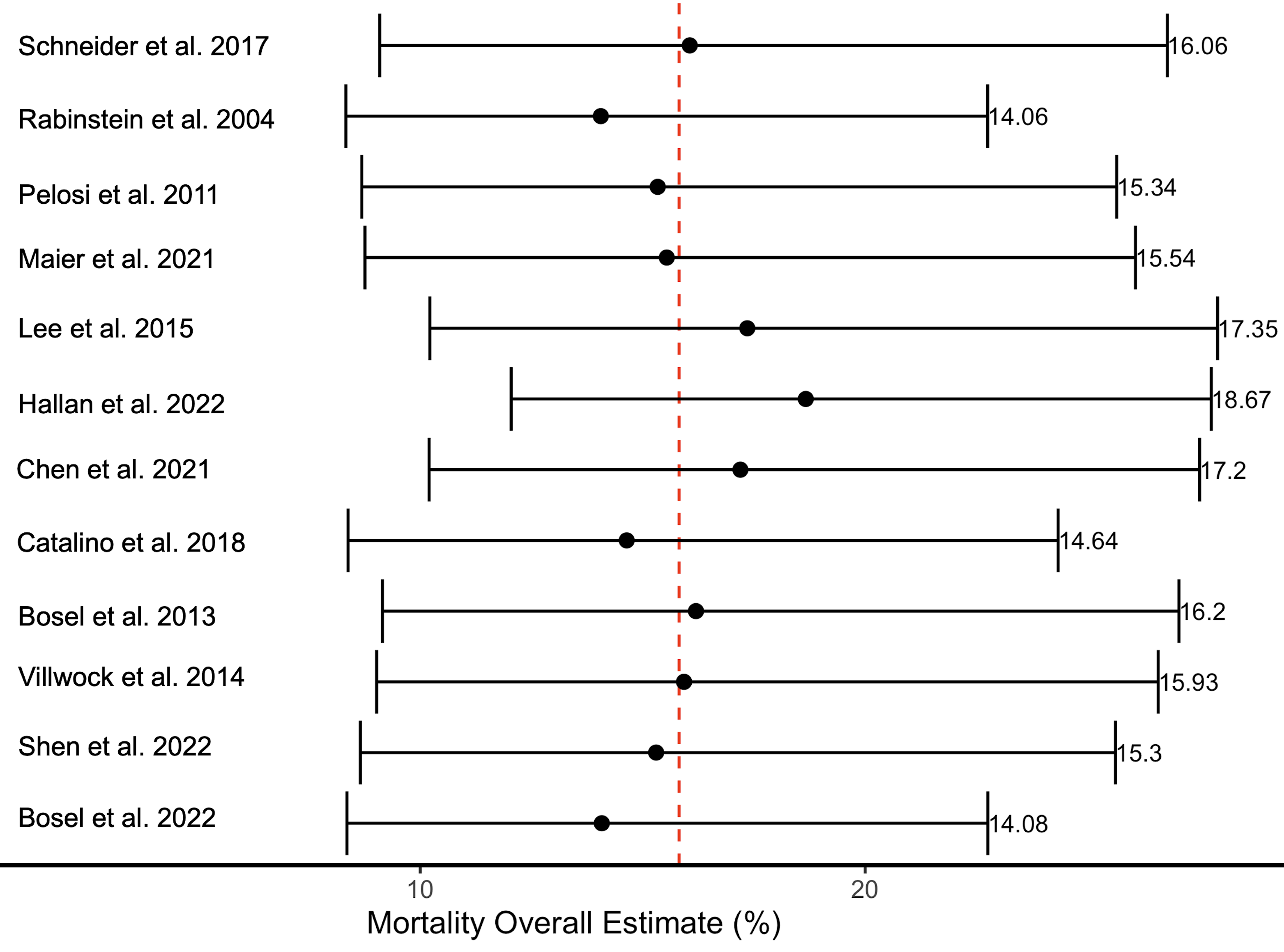


**Figure 2:** Cook’s Distance plot for meta-regression model for tracheostomy timing (mean time to tracheostomy) against mortality (%).


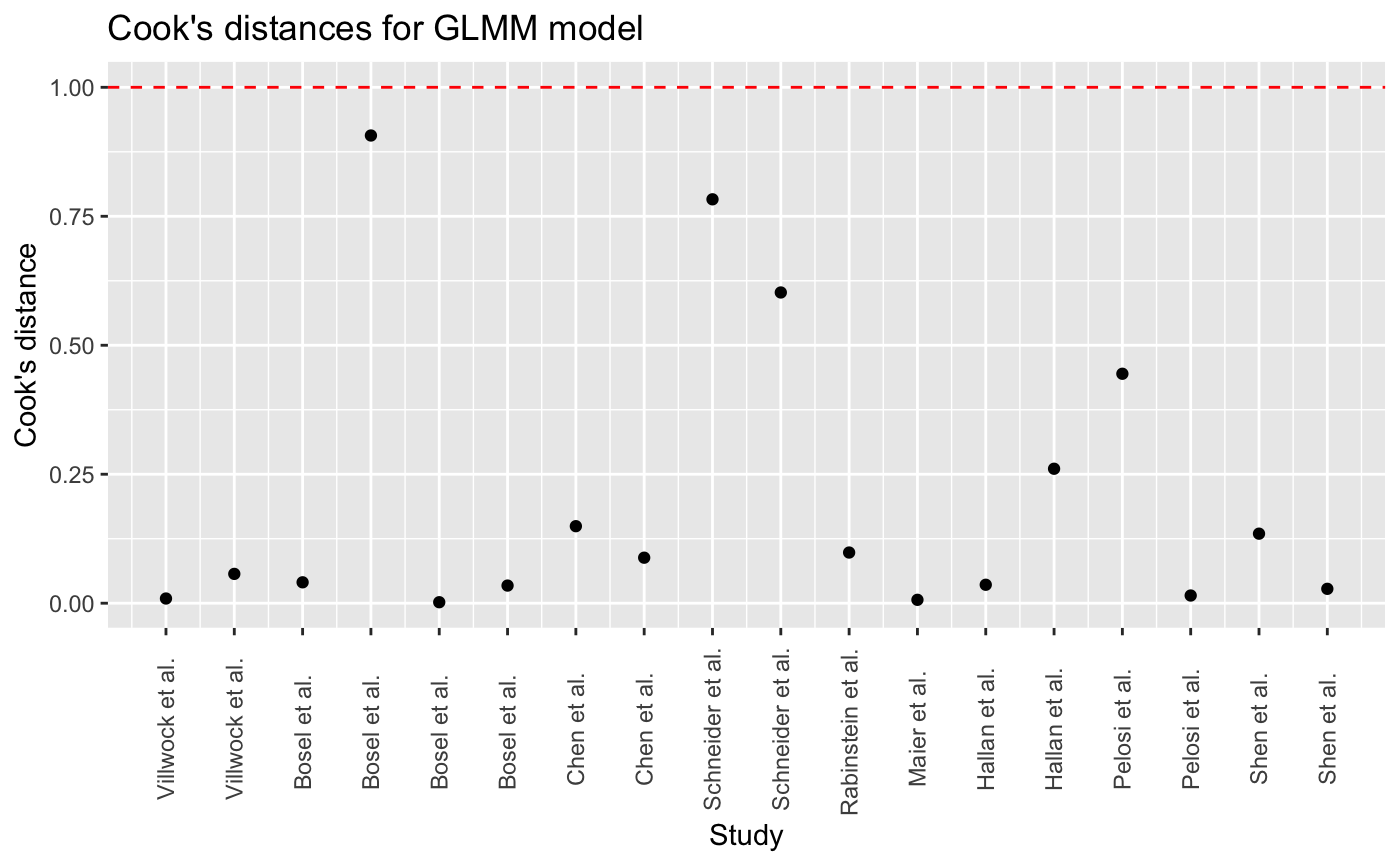


**ADDITIONAL FILE 1 – ITEM S3:** Outcomes reported (by study)

| **Study** | **Year** | **Stroke Proportion (IS: ICH)** | **SAH Inclusive** | **Mortality** | **Good Functional Outcome (Proportion)** | **mRS (Mean/Median Score)** | **VAP** | **ICU LOS** | **Hospital LOS** | **MV Days** | **Mean Time to Tracheostomy** |
| --- | --- | --- | --- | --- | --- | --- | --- | --- | --- | --- | --- |
| Alsherbini | 2019 | ✓ | ✓ |  |  |  |  | ✓ |  |  |  |
| Bosel et al. | 2013 | ✓ | ✓ | ✓ | ✓ | ✓ |  | ✓ |  | ✓ | ✓ |
| Bosel et al. | 2022 | ✓ | ✓ | ✓ | ✓ | ✓ |  | ✓ | ✓ | ✓ | ✓ |
| Catalino et al. | 2018 | ✓ |  | ✓ |  | ✓ | ✓ | ✓ | ✓ | ✓ |  |
| Chen et al. | 2019 | ✓ | ✓ | ✓ |  |  |  |  | ✓ |  | ✓ |
| Hallan et al. | 2022 | ✓ |  | ✓ |  |  |  | ✓ |  |  | ✓ |
| Lee et al. | 2015 | ✓ | ✓ | ✓ |  |  |  |  |  |  |  |
| Maier et al. | 2021 | ✓ |  | ✓ | ✓ | ✓ |  | ✓ | ✓ | ✓ | ✓ |
| Pelosi et al. | 2011 | ✓ |  | ✓ |  |  | ✓ | ✓ | ✓ | ✓ | ✓ |
| Rabinstein et al. | 2004 | ✓ | ✓ | ✓ | ✓ |  |  | ✓ | ✓ | ✓ | ✓ |
| Schneider et al. | 2017 | ✓ | ✓ | ✓ | ✓ | ✓ |  |  |  |  | ✓ |
| Shen et al. | 2022 | ✓ |  | ✓ |  |  | ✓ | ✓ | ✓ | ✓ | ✓ |
| Villwock et al. | 2014 |  |  | ✓ |  |  | ✓ |  | ✓ |  | ✓ |

**ADDITIONAL FILE 1 – ITEM S4:** Mean time to Tracheostomy (forest-plot)

**
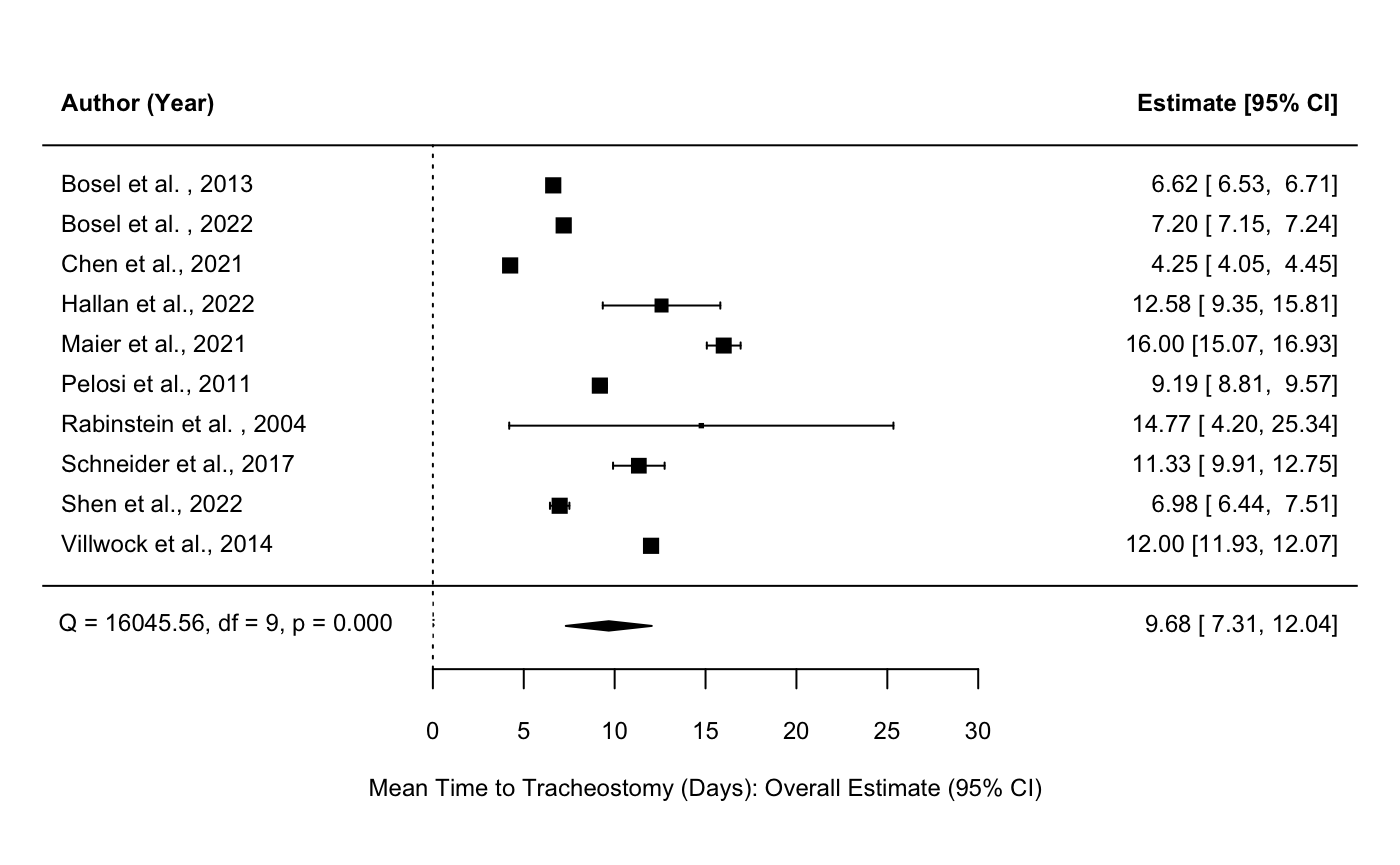
**

**ADDITIONAL FILE 1 – ITEM S5:** Ventilator Associated Pneumonia (forest-plot)


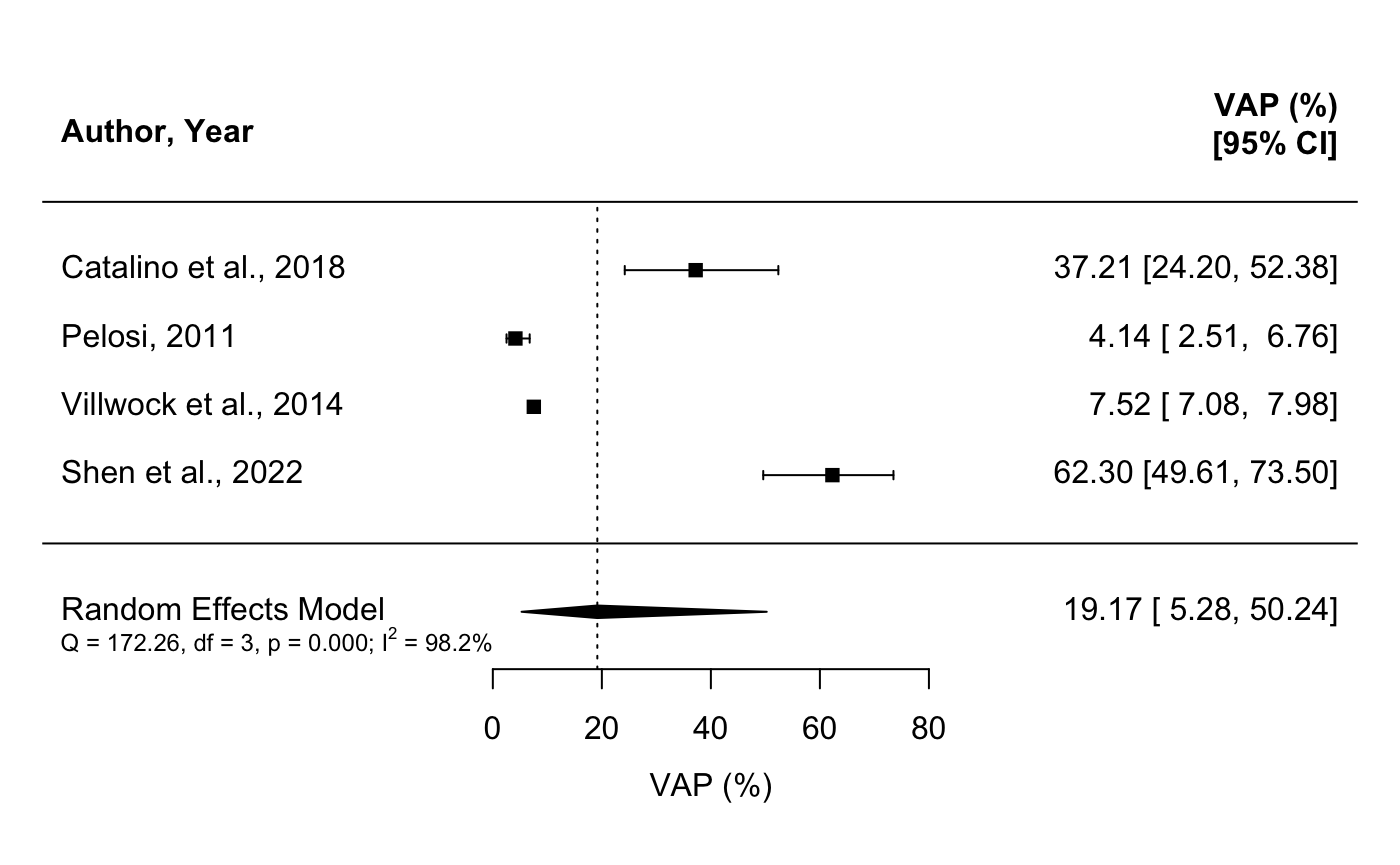


VAP was reported in 4 of the included studies^4,9,19,20^, overall prevalence was (19.17%, 95%CI = 5.28-50.24, p=<0.01, I^2^= 98.2%). Other complications included sepsis, reported by Bosel et al. 2013 (46.7%) and Schneider et al. 2017 (58.5%)

**ADDITIONAL FILE 1 – ITEM S6:** NOS study quality and bias assessment and Cochrane Risk of Bias Assessment Tool 2 (RoB 2)

| **Author** | **Year** | **Study Type** | **Setting** | **Selection** | **Comparability** | **Outcome** | **Total** |
| --- | --- | --- | --- | --- | --- | --- | --- |
| Alsherbini et al. | 2019 | Retrospective Cohort Study | Single-Center | **** | * | **** | 8 |
| Catalino et al. | 2018 | Retrospective Cohort Study | Single-Center | **** | ** | *** | 9 |
| Chen et al. | 2019 | Retrospective Cohort Study | Single-Center | **** | ** | *** | 9 |
| Hallan et al. | 2022 | Retrospective Cohort Study | Multi-Center | **** | * | *** | 8 |
| Lee et al. | 2015 | Retrospective Cohort Study | Single-Center | **** | * | *** | 8 |
| Maier et al. | 2021 | Retrospective Cohort Study | Single-Center | **** | * | *** | 8 |
| Pelosi et al. | 2011 | Retrospective Cohort Study | Multi-Center | **** | * | *** | 8 |
| Rabinstein et al. | 2004 | Retrospective Cohort Study | Single-Center | **** | * | *** | 8 |
| Schneider et al. | 2017 | Prospective Cohort Study | Single-Center | **** | * | *** | 8 |
| Shen et al. | 2022 | Retrospective Cohort Study | Single-Center | **** | * | *** | 8 |
| Villwock et al. | 2014 | Retrospective Cohort Study | Multi-Center | **** | * | *** | 8 |

*Average NOS was 8.2 ± 0.4 Therefore, studies were of good overall quality.*

**ADDITIONAL FILE 1 – ITEM S6 Continued:** Risk of Bias Assessment, Cochrane RoB 2

**
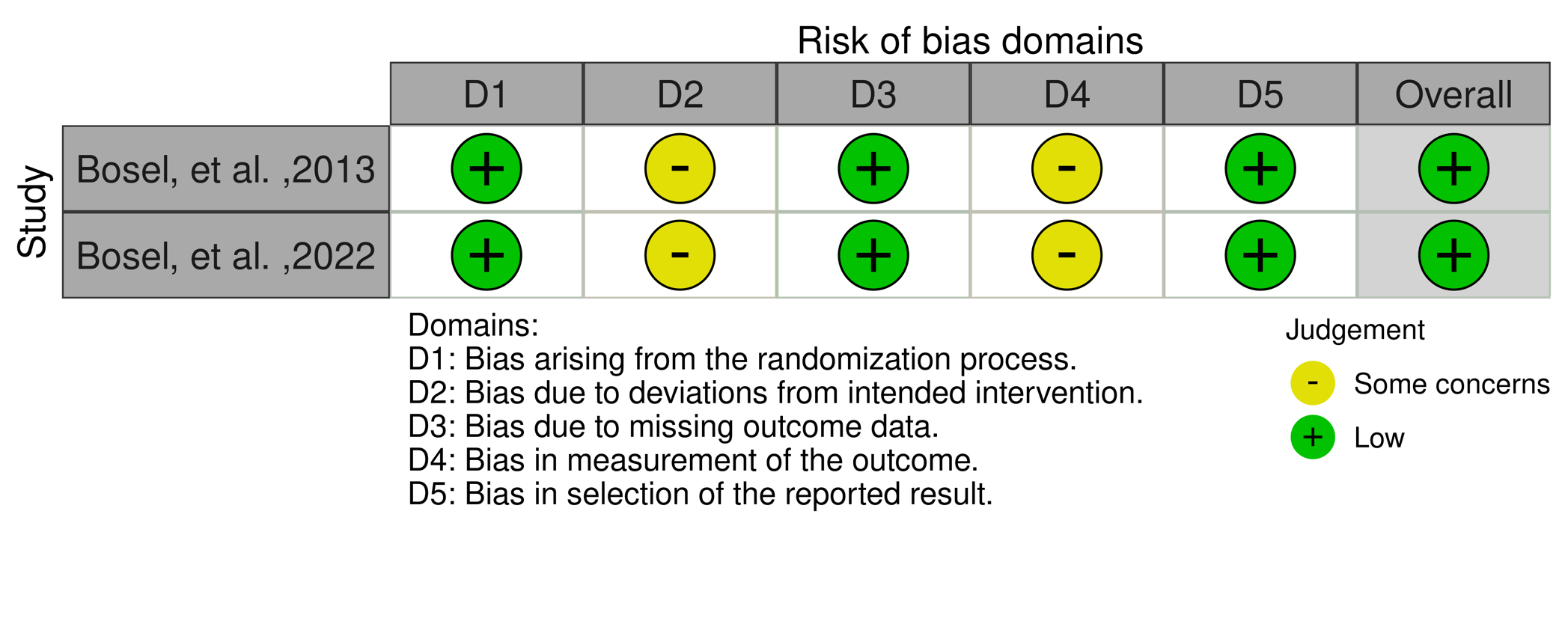
**

*Studies were assessed using RoB 2:*

Sterne JAC, Savović J, Page MJ, et al. RoB 2: a revised tool for assessing risk of bias in randomised trials. *BMJ*. 2019;366:l4898. doi:10.1136/bmj.l4898

**ADDITIONAL FILE 1 – ITEM S7:** Funnel plots & test of plot asymmetry

| **Variable** | **Limit Estimate/Intercept (b [95%CI])** | **p-value** | **Plot** |
| --- | --- | --- | --- |
| Mortality | 0.09 (-0.06-0.25) | 0.11 | 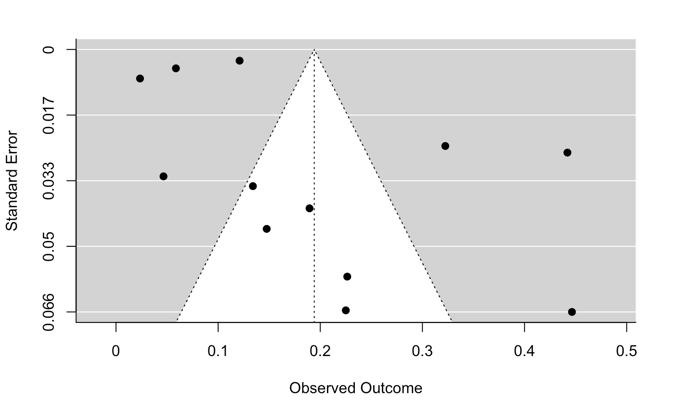 |
| Good Outcome (mRS 0-3) | 2.74 (0.42-5.04) | 0.31 | 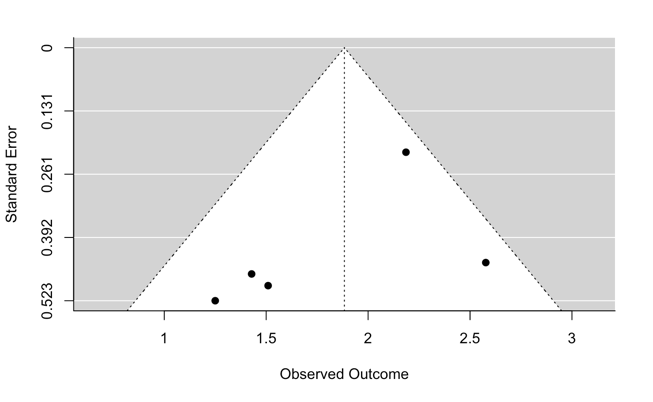 |
| Mean mRS | 4.84 (4.12-5.56) | 0.53 | 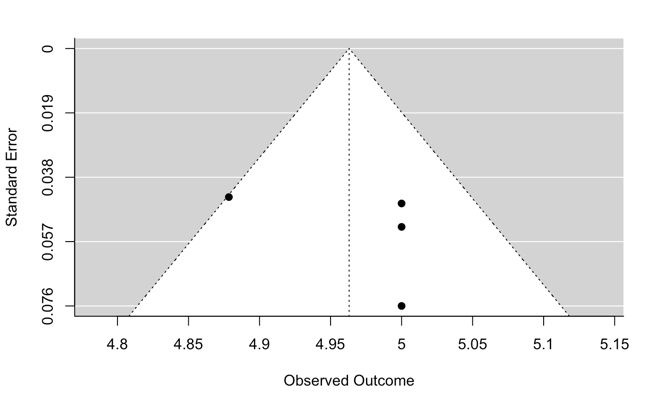 |
| MV Duration | 9.41 (-0.93-19.75) | 0.46 | 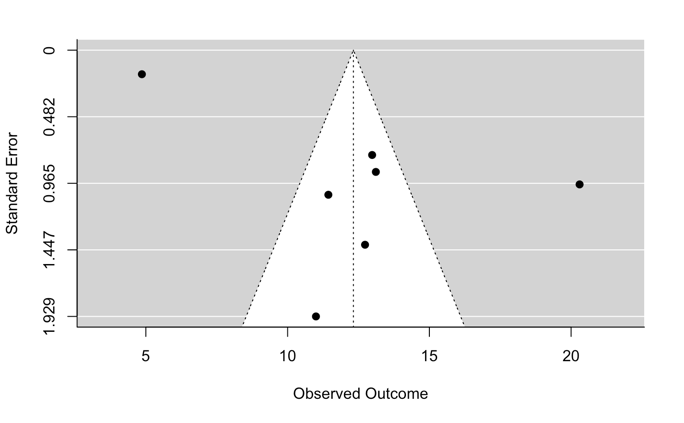 |
| Mean ICU LOS | 10.10 (1.95-18.26) | 0.08 | 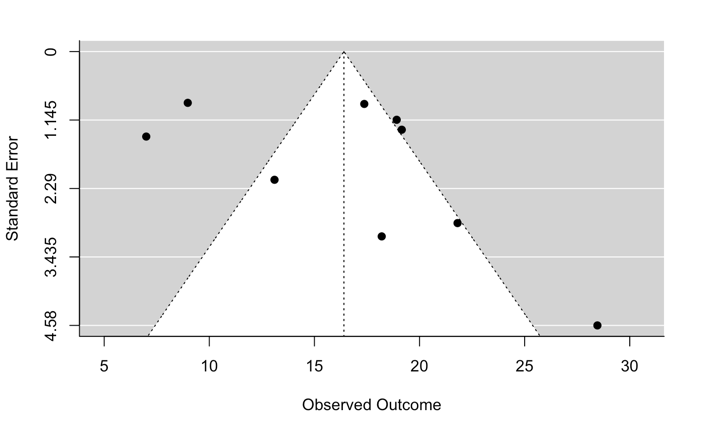 |
| Mean Hospital LOS | 26.34 (10.48-42.2) | 0.80 | 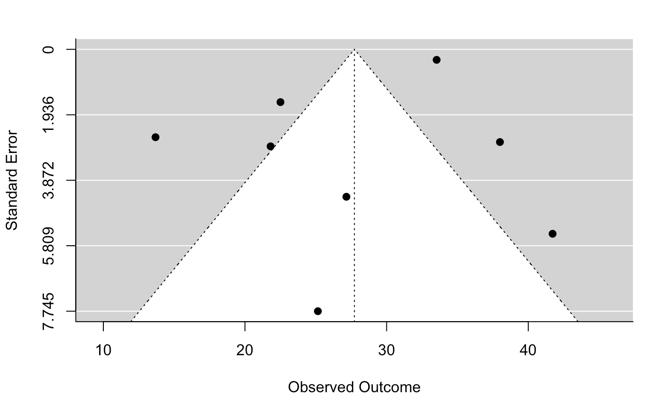 |
| Mean Time to Tracheostomy | 8.81 (5.89-11.72) | 0.19 | 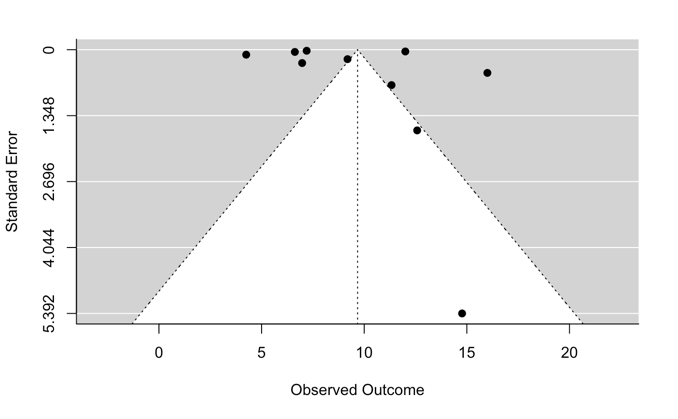 |

**ADDITIONAL FILE 1 – ITEM S8:** Unadjusted (A) and follow up adjusted overall mortality (B)

**
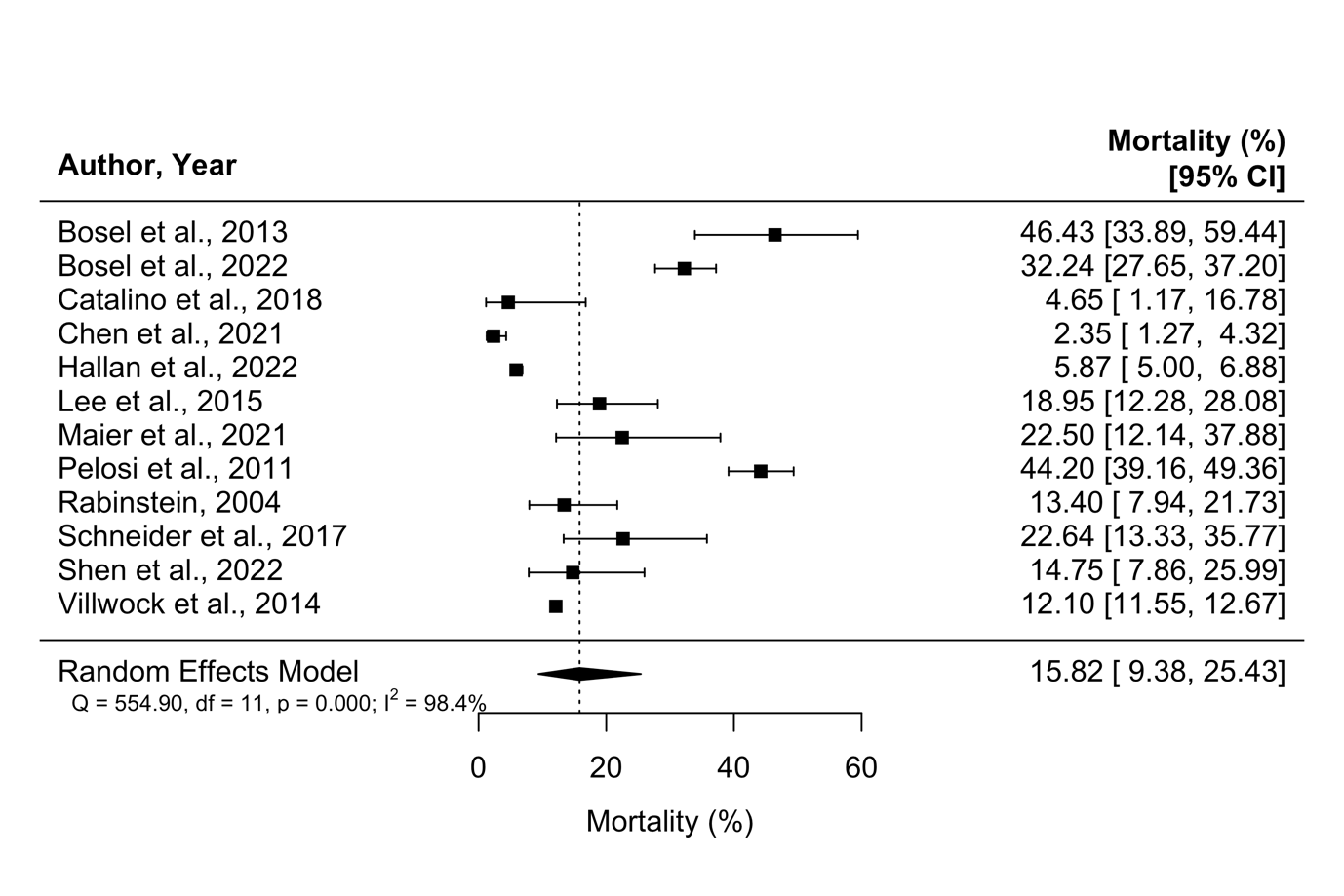
**

1. Follow Up Unadjusted Overall Mortality

**
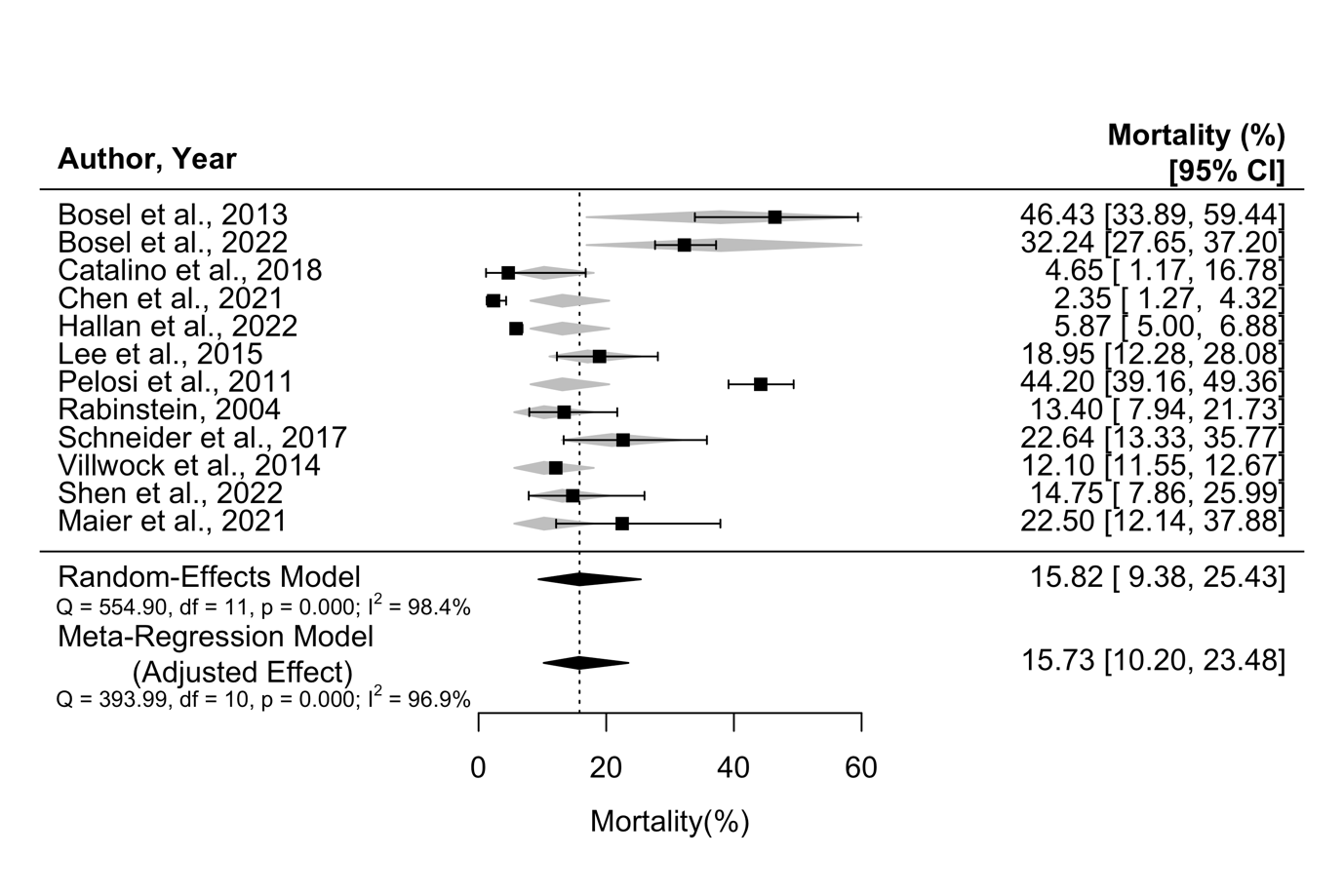
**

1. Follow Up Adjusted Overall Mortality


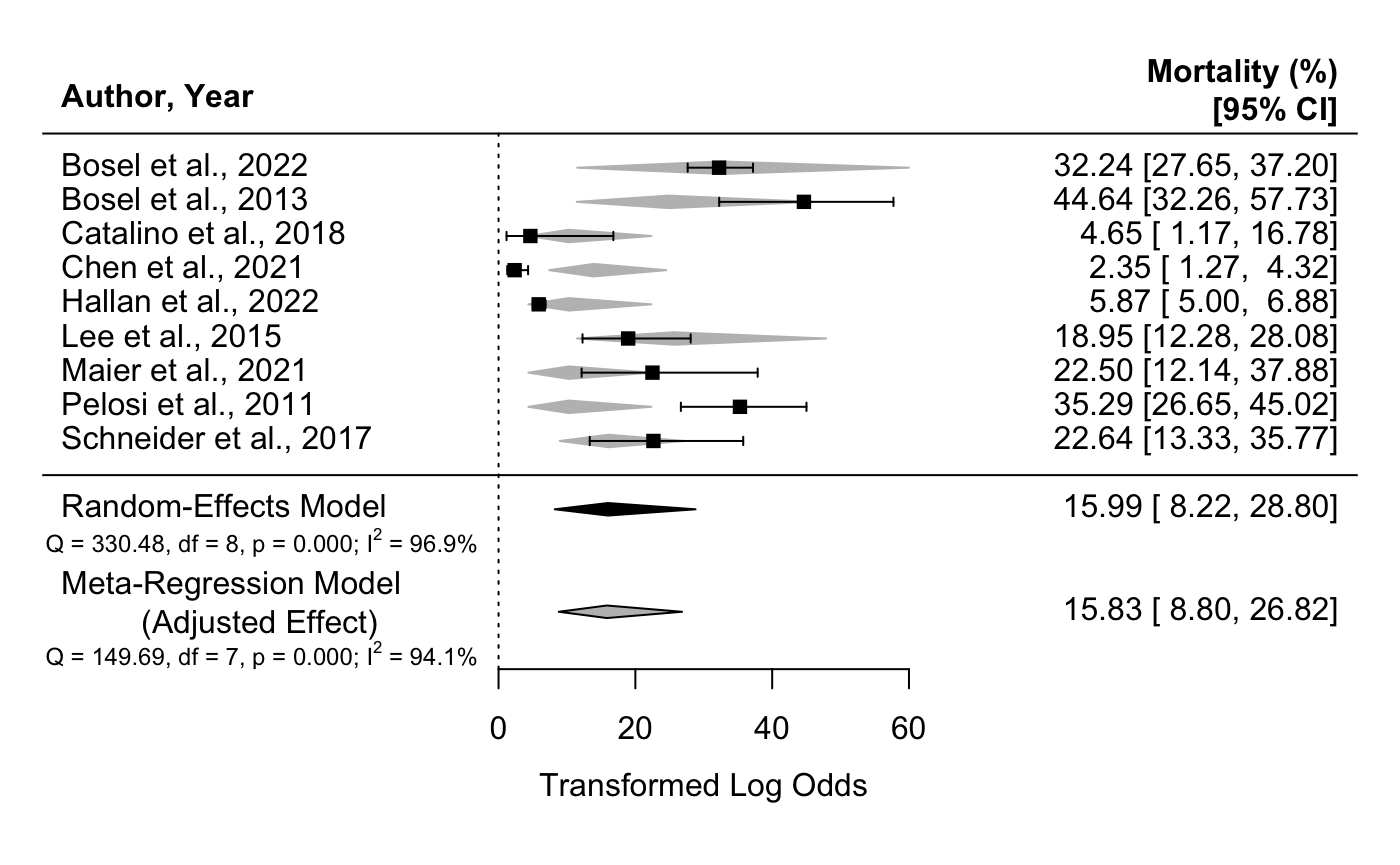


1. Overall Mortality (%) adjusted for % of patients with SAH.

**ADDITIONAL FILE 1 – ITEM S9:** ICU mortality overall estimate

|  | Mortality Estimate (%) | 95% CI | Number of studies (n) | Heterogeneity (I^2^, %) |
| --- | --- | --- | --- | --- |
| ICU-Mortality | 26.30 | 16.06-39.95 | 4 | 91.1 |

**ADDITIONAL FILE 1 – ITEM S10:** Multimodel Interference outputs with Information Criteria (AICc) and Weights (A-E)

| **Table A: Mortality** |  |  |  |  |  |  |
| --- | --- | --- | --- | --- | --- | --- |
|  | **Intercept** | **AIS:ICH** | **GCS on Admission** | **Mean Time to TT** | **AICc** | **delta** |
|  | + | -14.73 | 2.330 |  | 77.4 | 0.00 |
|  | + |  | 3.692 | 1.1170 | 77.5 | 0.08 |
|  | + | -13.65 | 3.749 | 0.9929 | 78.5 | 1.02 |
|  | + |  | 2.079 |  | 79.5 | 2.03 |
|  | + | 19.63 |  | -0.1490 | 115.8 | 38.40 |
| **Table B: Good mRS** |  |  |  |  |  |  |
|  | **Intercept** | **AIS:ICH** | **GCS on Admission** | **Mean Time to TT** | **AICc** | **delta** |
|  | + |  | -0.3271 |  | 51.0 | 0.00 |
|  | + | -29.37 |  |  | 53.6 | 2.62 |
|  | + |  |  | -0.2137 | 59.4 | 8.40 |
|  | + |  |  |  | 60.5 | 9.46 |
|  | + | -27.04 |  | -0.1958 | 60.6 | 9.62 |

| **Table C: Mean mRS** |  |  |  |  |  |  |
| --- | --- | --- | --- | --- | --- | --- |
|  | **Intercept** | **AIS:ICH** | **GCS on Admission** | **Mean Time to TT** | **AICc** | **delta** |
|  | + |  |  |  | 17.5 | 0.00 |
|  | + |  |  | 0.01925 | 23.5 | 6.06 |
|  | + | 0.6895 |  |  | 23.5 | 6.07 |
|  | + |  | -0.01379 |  | 26.6 | 9.14 |
|  | + | 0.4891 |  | 0.01377 | 36.5 | 19.02 |
|  |  |  |  |  |  |  |

| **Table D: Mean MV Days** |  |  |  |  |  |  |
| --- | --- | --- | --- | --- | --- | --- |
|  | **Intercept** | **AIS:ICH** | **GCS on Admission** | **Mean Time to TT** | **AICc** | **delta** |
|  | + |  | 2.239 |  | 43.7 | 0.00 |
|  | + | 2.243 | 2.073 |  | 48.5 | 4.77 |
|  | + |  | 2.180 | -0.04769 | 48.6 | 4.92 |
|  | + | 7.716 |  |  | 52.1 | 8.42 |
|  |  |  |  |  |  |  |

| **Table E: Mean ICU LOS** | |  |  |  |  |  |  |
| --- | --- | --- | --- | --- | --- | --- | --- |
|  |  | **Intercept** | **AIS:ICH** | **GCS Admission** | **Mean Tt** | **AICc** | **delta** |
|  |  | + |  | 1.960 |  | 47.9 | 0.00 |
|  |  | + |  | 2.374 | 0.3339 | 51.8 | 3.95 |
|  |  | + | 2.716 | 1.769 |  | 52.0 | 4.16 |
|  |  | + | 6.321 |  | 0.3239 | 61.6 | 13.71 |
|  |  | + | 5.841 |  |  | 62.3 | 14.39 |
|  |  |  |  |  |  |  |  |

*Models were excluded from results/interpretation: models with the least number of moderators are optimal (parsimony). These models are likely to be overfitted and clinically irrelevant. Only the top 5 models are presented and from this, relative importance value calculated.

**ADDITIONAL FILE 1 – ITEM S11:** Proportion of good neurological outcome (mRS 0-3, %), Mechanical Ventilation Duration, Hospital Length of Stay, ICU-Length of stay (forest-plot, A-D)


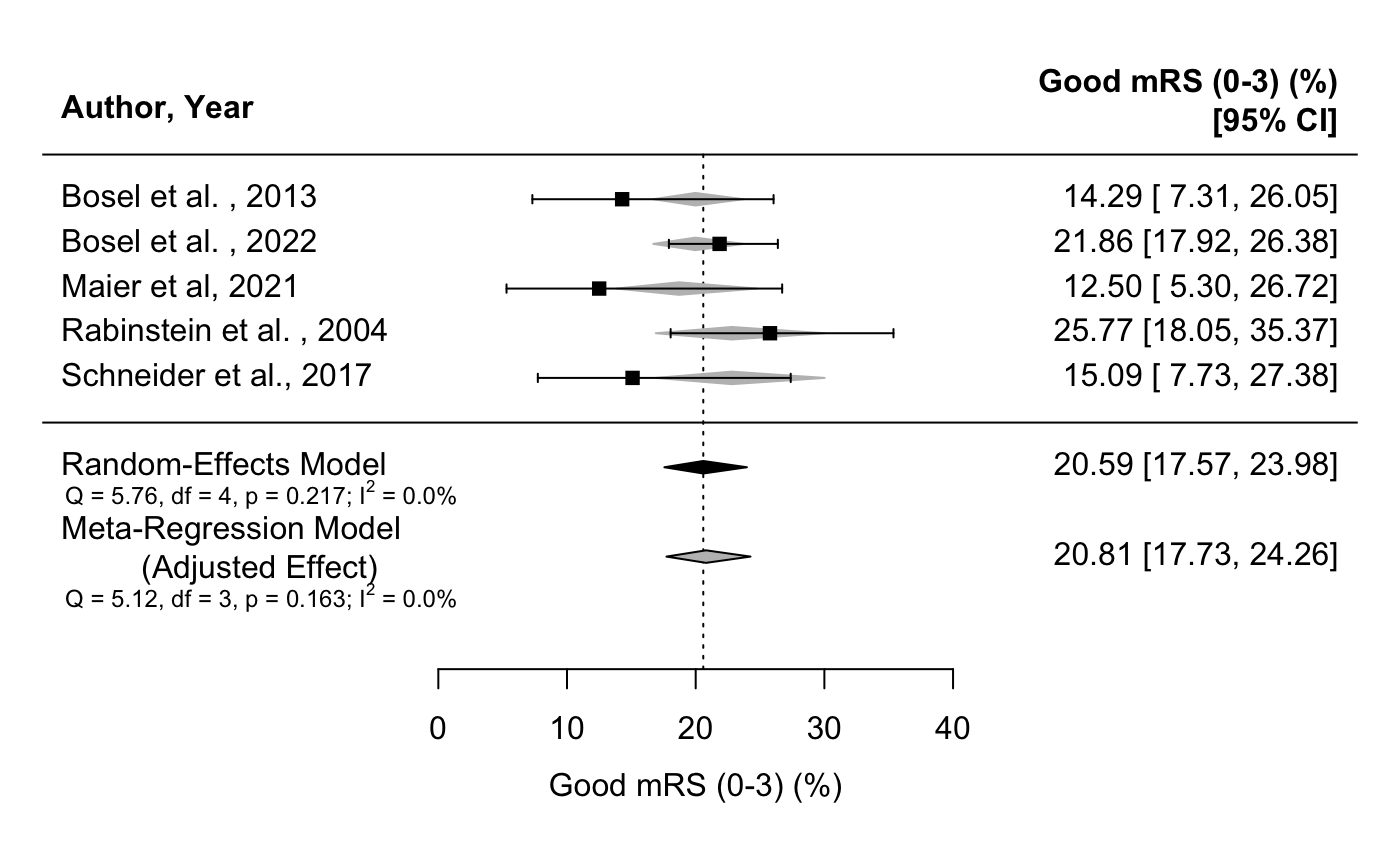


1. Overall Good mRS Follow Up Adjusted (Proportion)

**
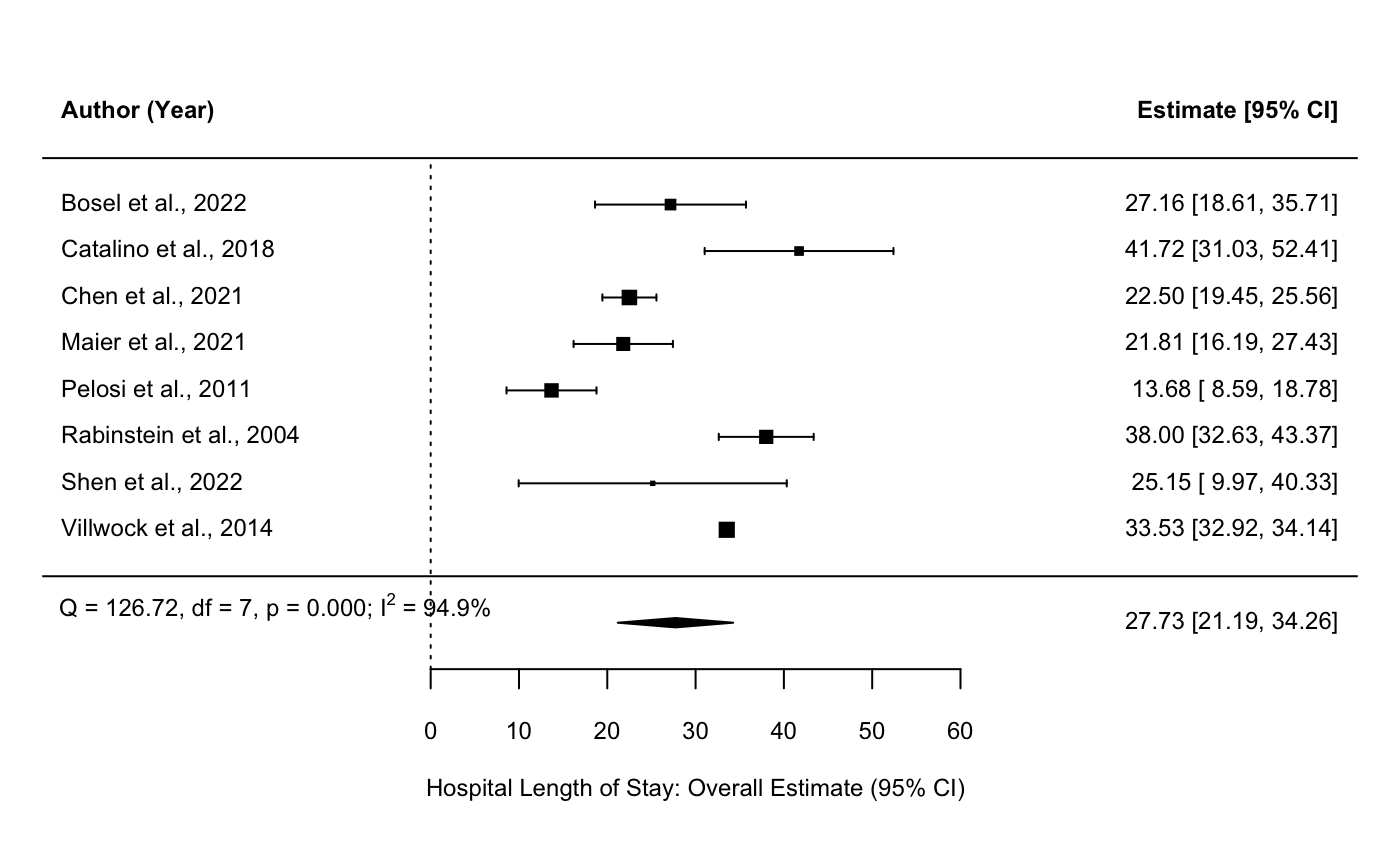
**

1. Hospital Length of Stay

**
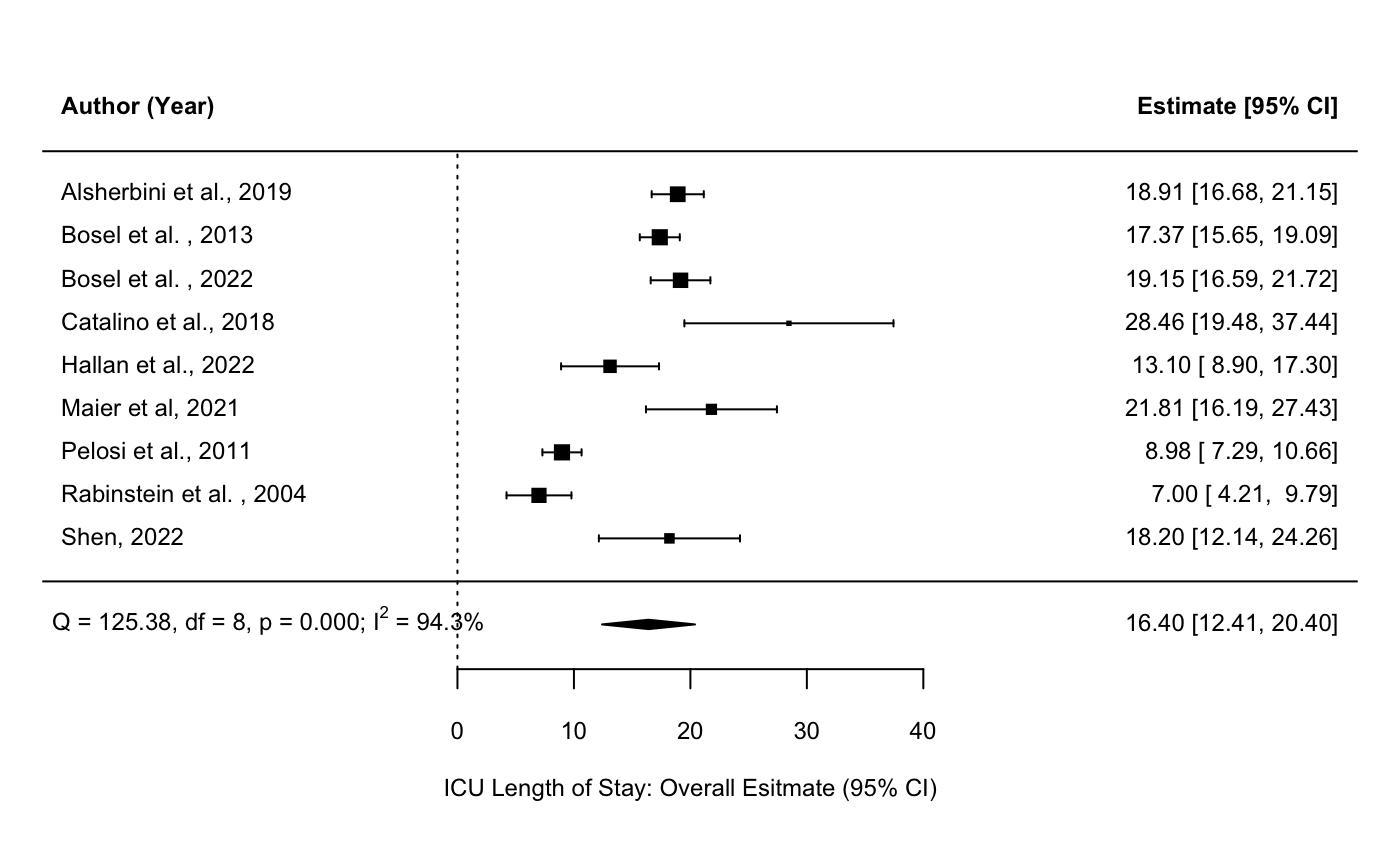
**

1. ICU Length of Stay

**
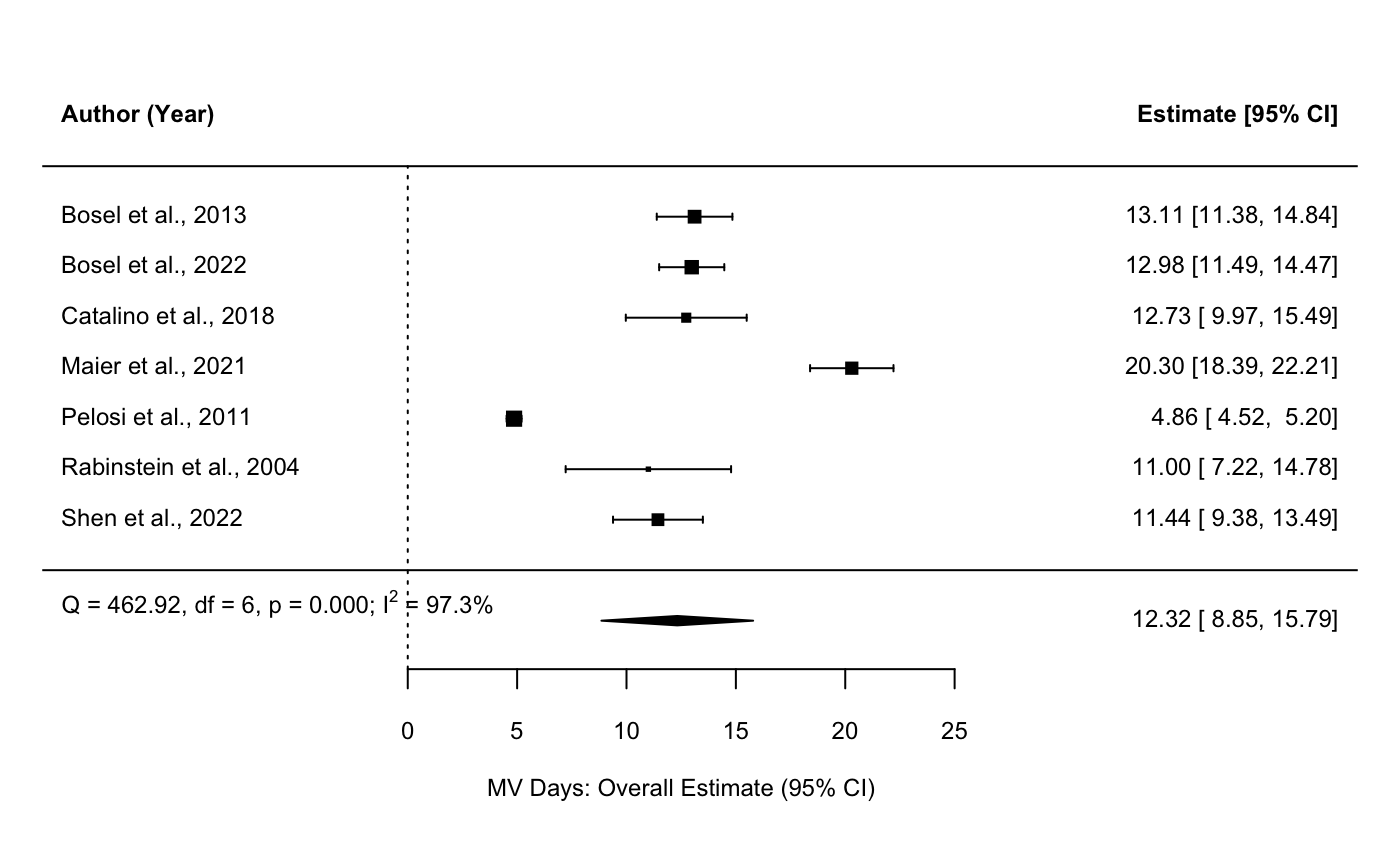
**

1. Mechanical Ventilation Duration

**ADDITIONAL FILE 1 – ITEM S12:** Meta-regression outputs

|  |  | Estimate | 95% CI | p-value | Plot |
| --- | --- | --- | --- | --- | --- |
| **Mortality** | | | | | |
|  | MeanTt | -0.28 | -2.30- 1.74 | 0.77 | 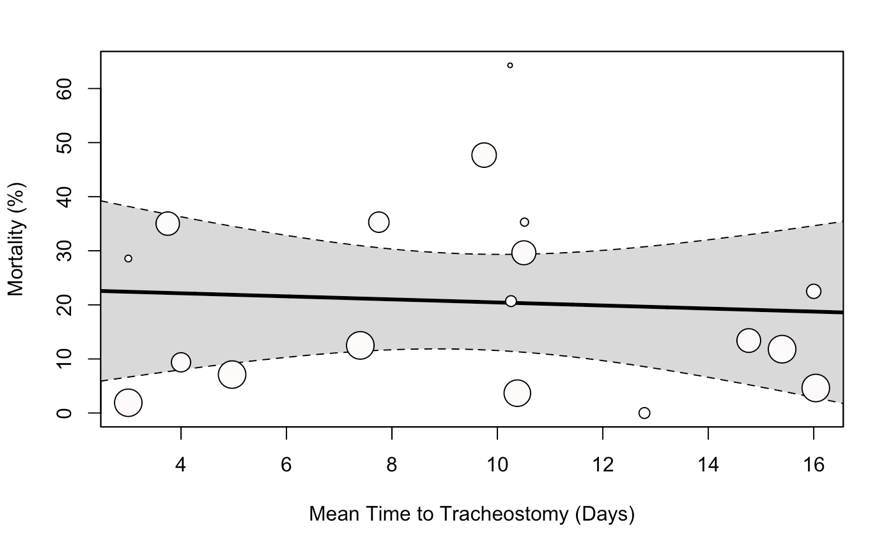 |
|  | Intercept | 23.30 | 2.18-44.35 | 0.03* |  |
| **Good Functional Outcome** | | | | | |
|  | MeanTt | -0.21 | -2.62-2.19 | 0.84 | 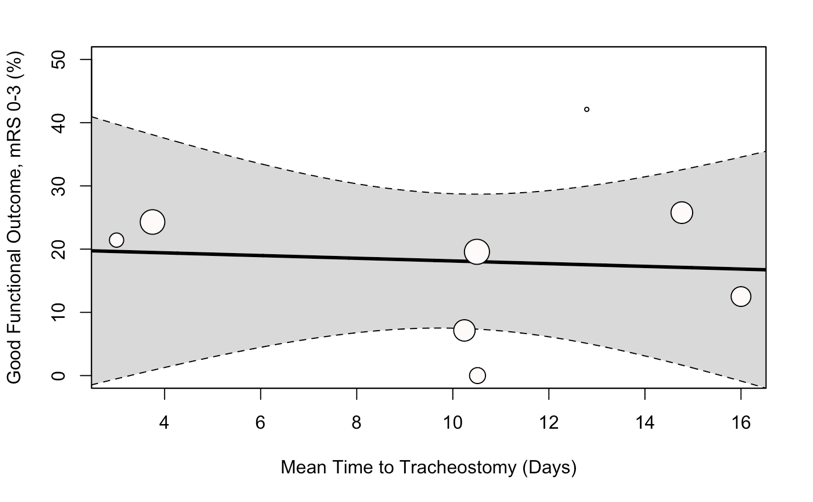 |
|  | Intercept | 20.27 | -6.26-46.8 | 0.11 |  |
| **Mean mRS** |  |  |  |  |  |
|  | MeanTt | 0.02 | -0.12-0.16 | 0.74 | 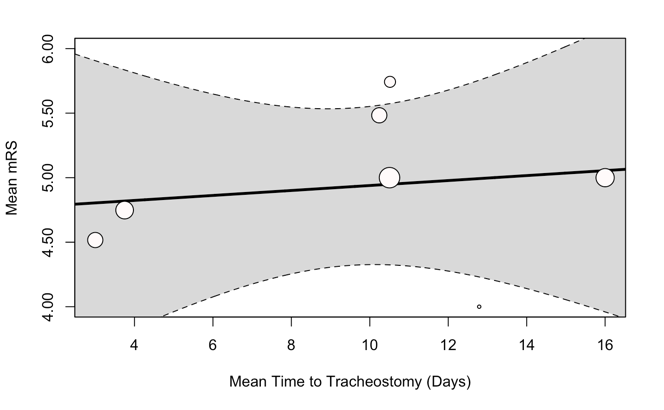 |
|  | Intercept | 4.75 | 3.27-6.21 | <0.01* |  |

| **Mean MV Duration** | | | | | |
| --- | --- | --- | --- | --- | --- |
|  | MeanTt | 0.27 | -0.56-1.10 | 0.48 | 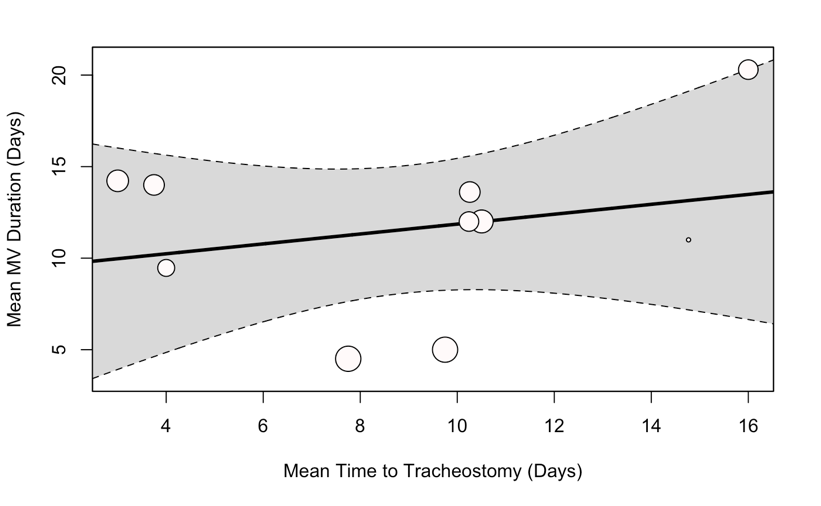 |
|  | Intercept | 9.16 | 0.94-17.38 | 0.03 |  |
| **Mean ICU LOS** | | | | | |
|  | MeanTt | 0.03 | -0.76-0.82 | 0.94 | 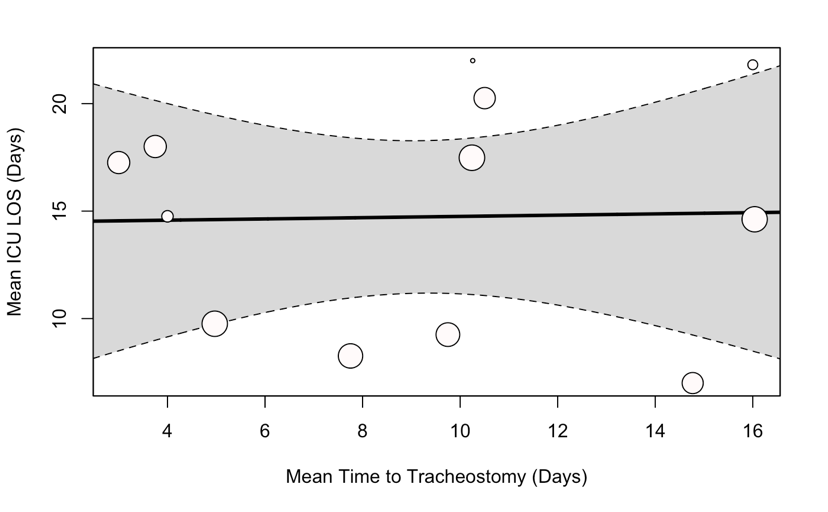 |
|  | Intercept | 14.46 | 6.37-22.54 | <0.01 |  |

| **Mean HLOS** |  |  |  |  |  |
| --- | --- | --- | --- | --- | --- |
|  | MeanTt | 0.81 | -0.68-2.31 | 0.0404 | 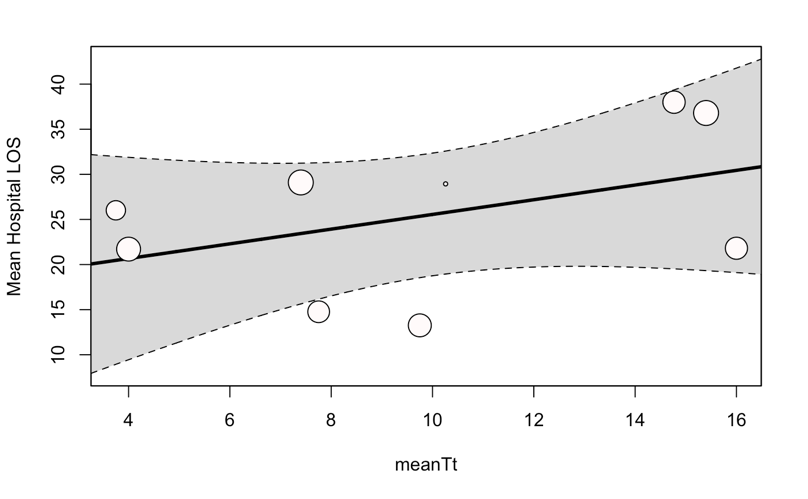 |
|  | Intercept | 17.4 | 1.01-33.8 | 0.04 |  |

**ADDITIONAL FILE 1 – ITEM S13:** Additional Results (Mean mRS score)

Additional Results; Mean mRS score

We observed no association between mean mRS score and time to tracheostomy (β= 0.02, 95% CI =-0.12-0.16, p=0.74, **Additional file ITEM S12**). Mean time to tracheostomy did not account for any heterogeneity observed in mean mRS score (R^2^=0.00% p<0.01). Addition of AIS:ICH ratio and GCS on admission to the mixed effects meta regression model did not offer further clarity.

**ADDITIONAL FILE 1 – ITEM S14:** Additional Results and Discussion (Mean MV Duration)

**Mechanical Ventilation Duration**

Mean MV duration was 12.32 days (95%CI=8.85-15.79, I^2^=97.3%, **Additional file ITEM S11D**). MV Duration was not associated with time to tracheostomy (β= 0.27, 95%CI =-0.56-1.10, p=0.48 **(Additional file ITEM S12)**. Limited data prevented multiple moderators from being included in the meta-regression. Study year (alone) and GCS on admission (alone) accounted for 35.71% and 13.43% of between study heterogeneity respectively. Significant heterogeneity remained in both instances (p<0.01).

**Discussion of Findings (MV Duration)**

Patients with earlier tracheostomy are hypothesized to have decreased length of sedation and earlier mobilization^2,45^. However, these benefits are variable: meta-analysis of RCTs in critical ill patients found (in 2015) no difference in duration or MV between early and late groups^46^. On the other hand Deng et al., (2021) reported that patients with early (1-8 days) tracheostomy (versus late; 6-28 days) had shorter MV duration (standardised mean difference: –1.17; 95% CI: –2.10 to –0.24; P =0.014)^40^. This finding was not maintained upon trial sequential analysis (specifically in RCTs with younger patients)^40,47^. In critically ill stroke patients, the SETPOINT-2 trial concluded that benefits of early tracheostomy are outweighed by longer weaning duration^3^. We corroborated this finding. Therefore, it is likely that severe injury complicated by unresolved respiratory failure or difficulty weaning lengthens MV duration even in patients who receive early tracheostomy. Indeed, in the observational setting, the initial indication for earlier tracheostomy is often more severe disease. Unsurprisingly, our analysis found that more traditional indicators of disease severity (GCS on admission, stroke type) modelled MD duration better than tracheostomy timing.

**ADDITIONAL FILE 1 – ITEM S15:** SETPOINT-2 threshold interaction term outputs (Mortality and ICU-LOS)

Threshold Test based on SETPOINT-2 Definition (Early: <5 days, Late: ≥10 days) For mortality (6A) and ICU length of stay (6B)

**Table A:** Mortality vs. Mean Time to Tracheostomy

|  | **Estimate** | **95% CI** | **p-value** |
| --- | --- | --- | --- |
| <5days | 0.72 | -22.32-23.75 | 0.95 |

**Table B:** Mean ICU LOS vs. Mean Time to Tracheostomy

|  | **Estimate** | **95% CI** | **p-value** |
| --- | --- | --- | --- |
| <5days | -2.38 | -10.02-5.27 | 0.44 |

Threshold tests were performed using the interaction term mean time to tracheostomy * binary moderator (early vs. late, SETPOINT-2 definition). The test was preformed when at least 3 studies in each group were present. Here we determined that there was no significant difference between the mortality for studies with mean time to tracheostomy less than 5 days compared to those with tracheostomy timing greater than 10 days.

**ADDITIONAL FILE 1 – ITEM S16:** Sensitivity analysis (Mortality): Early vs. Late tracheostomy (subgroup)


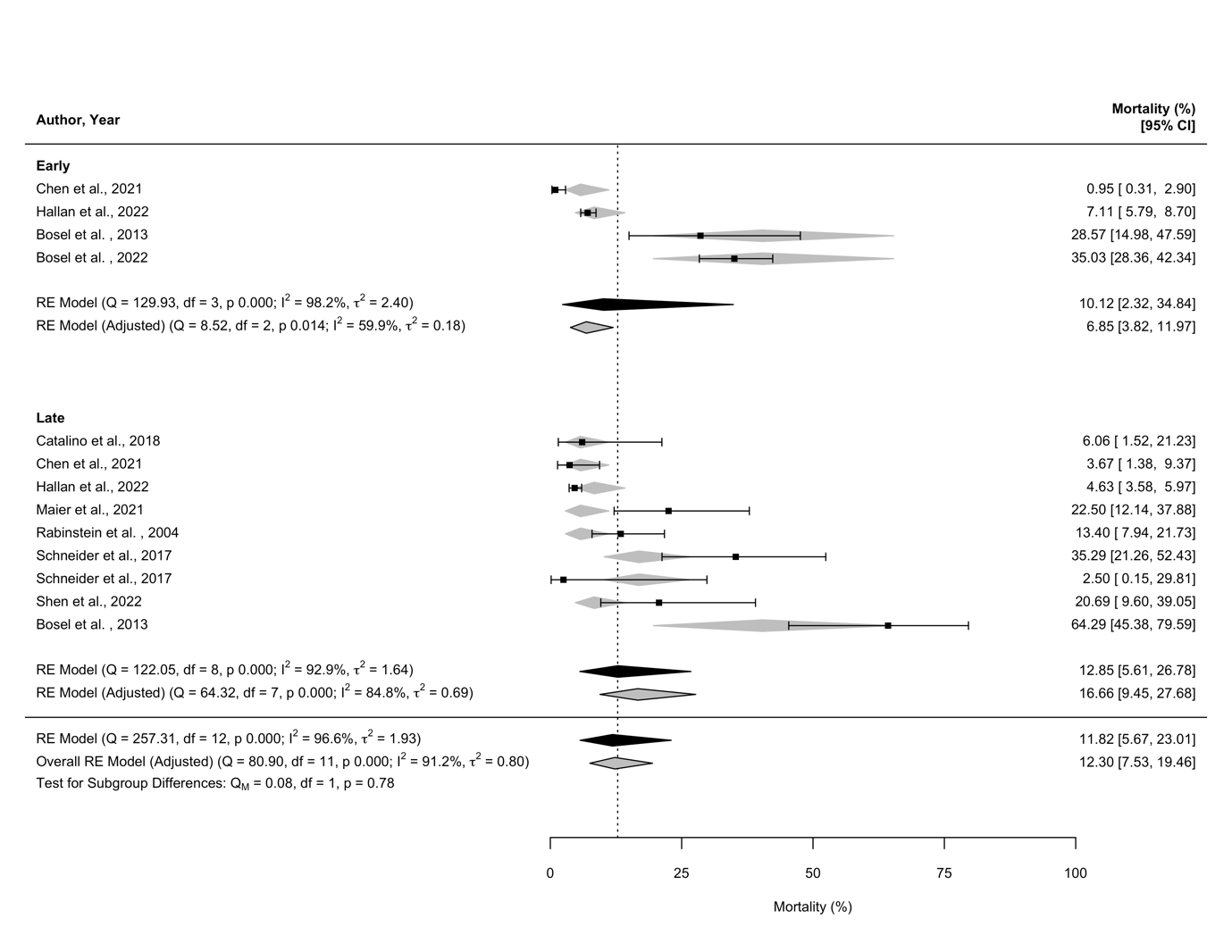


**ADDITIONAL FILE 1 – ITEM S17:** Test of correlation between moderator variables

**
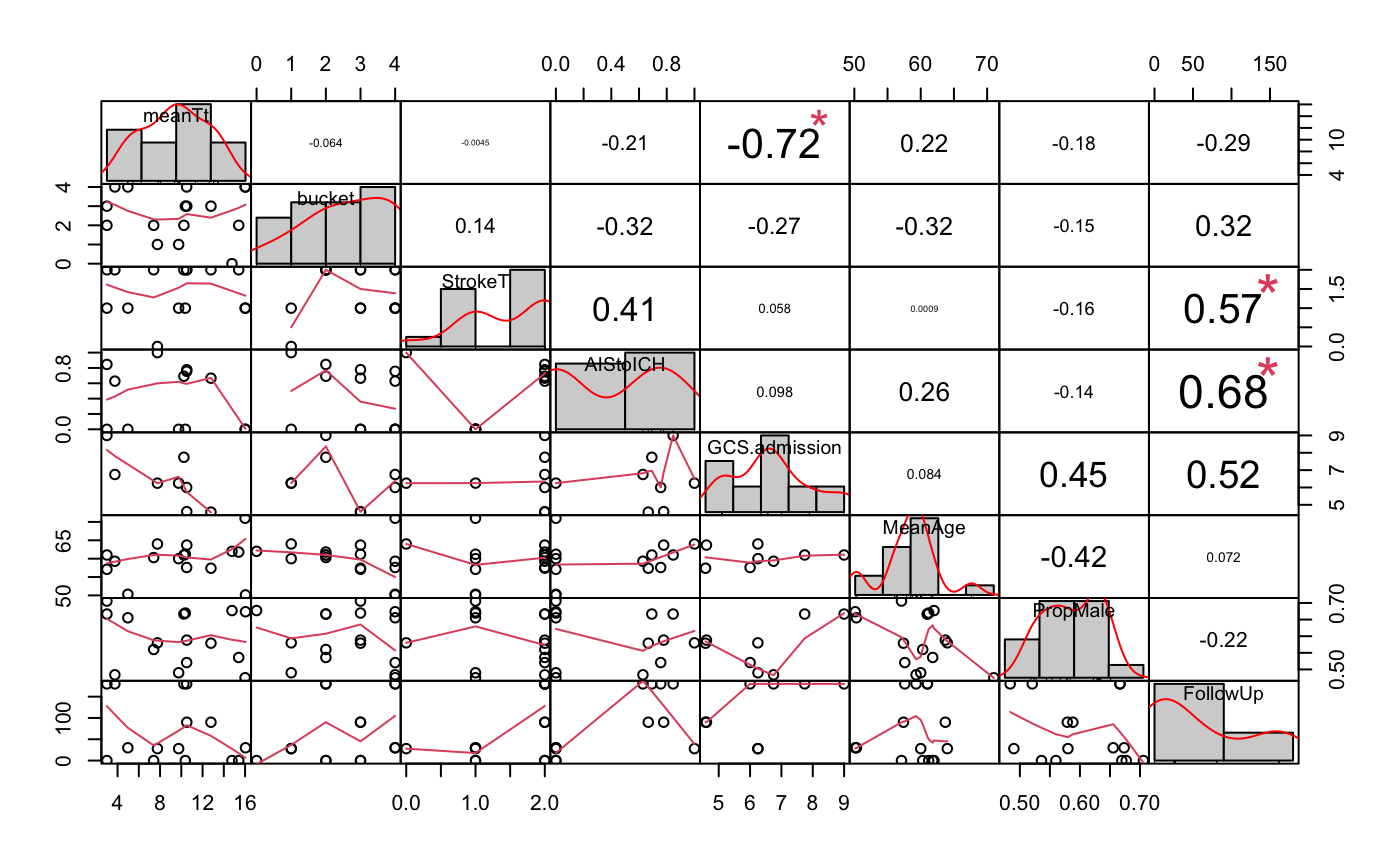
**
